# Bourdieu’s Cultural Capital in Relation to Food Choices: A Systematic Review of Cultural Capital Indicators and an Empirical Proof of Concept

**DOI:** 10.1371/journal.pone.0130695

**Published:** 2015-08-05

**Authors:** Carlijn B. M. Kamphuis, Tessa Jansen, Johan P. Mackenbach, Frank J. van Lenthe

**Affiliations:** Department of Public Health, Erasmus University Medical Centre, Rotterdam, The Netherlands; Hunter College, UNITED STATES

## Abstract

**Objective:**

Unhealthy food choices follow a socioeconomic gradient that may partly be explained by one’s ‘cultural capital’, as defined by Bourdieu. We aim 1) to carry out a systematic review to identify existing quantitative measures of cultural capital, 2) to develop a questionnaire to measure cultural capital for food choices, and 3) to empirically test associations of socioeconomic position with cultural capital and food choices, and of cultural capital with food choices.

**Design:**

We systematically searched large databases for the key-word ‘cultural capital’ in title or abstract. Indicators of objectivised cultural capital and family institutionalised cultural capital, as identified by the review, were translated to food choice relevant indicators. For incorporated cultural capital, we used existing questionnaires that measured the concepts underlying the variety of indicators as identified by the review, i.e. participation, skills, knowledge, values. The questionnaire was empirically tested in a postal survey completed by 2,953 adults participating in the GLOBE cohort study, The Netherlands, in 2011.

**Results:**

The review yielded 113 studies that fulfilled our inclusion criteria. Several indicators of family institutionalised (e.g. parents’ education completed) and objectivised cultural capital (e.g. possession of books, art) were consistently used. Incorporated cultural capital was measured with a large variety of indicators (e.g. cultural participation, skills). Based on this, we developed a questionnaire to measure cultural capital in relation to food choices. An empirical test of the questionnaire showed acceptable overall internal consistency (Cronbach’s alpha of .654; 56 items), and positive associations between socioeconomic position and cultural capital, and between cultural capital and healthy food choices.

**Conclusions:**

Cultural capital may be a promising determinant for (socioeconomic inequalities in) food choices.

## Introduction

Poor dietary intake is a major risk factor for morbidity and mortality [1,2]. Compared to high socioeconomic groups, low socioeconomic groups typically engage more in unhealthy behaviours, such as unhealthy food choices [3–8]. Therefore, the behavioural explanation is an important mechanism underlying socioeconomic inequalities in health [9–11]. Our understanding of *why* low socioeconomic groups typically engage in unhealthy behaviours is still insufficient [12].

Unhealthy behaviours are thought not to be the result of entirely voluntary choices, but rather influenced by structures in the daily context, such as material resources [13]. Material deprivation, which is related to a lower household income and poorer living conditions (i.e. poorer housing conditions, more financial problems), has been shown to partly mediate the association between socioeconomic position and health behaviours [13]. However, disparities in material resources are not the only explanation. For instance, a robust finding in health inequalities research is the social gradient, which illustrates that poorer health and health behaviours are not only found among the worst off; a surplus in income relates to a better health status at *each* level of the social hierarchy, also when comparing the highest with the second-highest income level [14]. Similarly, healthy dietary intake also continues to improve above the level of poverty [3].

Health behaviours may also depend on one’s sociocultural resources. By means of socialisation processes, dispositions and behaviours of individuals become similar within each social class and generation[15]. The influential French sociologist Pierre Bourdieu described how health- and lifestyle behaviours may therefore be subject to class distinction; high socioeconomic groups differentiate themselves from low socioeconomic groups by adopting healthy lifestyles, and low socioeconomic groups distinguish themselves from high socioeconomic groups with behaviours that give them a sense of ‘freedom from convention’ [16]. Bourdieu depicted how high socioeconomic groups distinguish themselves from low socioeconomic groups through their ‘taste’ for various lifestyle attributes, such as musical, artistic and culinary tastes [17]. He states that taste is developed through *cultural capital*, a non-material resource that accumulates throughout the life course. Although taste is also linked to economic capital, it mainly relates to cultural capital, as taste remains stable, also when people’s income increases over time, thereby reflecting certain cultural norms and values [17,18].

Possession of cultural capital may thus be a missing link in the relation between socioeconomic position and food choice behaviour. Although the relation between cultural capital and health, healthy lifestyles, and health behaviours is a subject of growing interest [19–24], the concept has found little empirical application in studies regarding socioeconomic inequalities in health behaviours so far (e.g. [23,25]). Likely, research into the role of cultural capital is hampered by the unfamiliarity of researchers with the concept, and by the lack of clear indicators and measures of cultural capital that can be applied to study (inequalities in) health behaviours. Abel (2008) noted that new indicators need to be developed for studying cultural capital in relation to health and health behaviour [20].

The aim of the present paper was to develop a set of questionnaire items in order to investigate to what extent the possession of cultural capital differs between socioeconomic groups, and to what extent cultural capital relates to healthy and unhealthy food choices. We first wanted to learn about currently used measures of cultural capital. Therefore, (1) we systematically reviewed the literature in order to identify currently used quantitative measures of cultural capital. Subsequently, based on these findings (2) we developed a set of questionnaire items to study cultural capital in relation to food choices. Finally, (3) we piloted our questionnaire to assess its applicability in empirical research, and investigated a) associations of socioeconomic position (i.e. respondent’s highest attained educational level) with unhealthy food choices (adjusted for age and sex), b) associations of socioeconomic position with cultural capital, and c) associations of cultural capital with unhealthy food choices (adjusted for socioeconomic position, age and sex).

First, we will give an introduction into the concept of cultural capital, the three forms of cultural capital, and its relation to economic and social capital.

### Cultural capital theory

The cultural capital notion provides an explanation of social stratification mechanisms and was originally conceptualised by Bourdieu to explain class differences in academic achievement [26–28]. Cultural capital is acquired through education and socialisation and includes “the distinctive forms of knowledge and ability that students acquire […] from their training in the cultural disciplines” (Bourdieu, 2010, p. xviii) [17]. Through available cultural capital in the family, one is more inclined to ‘inherit’ cultural resources that can be mobilised to accumulate incorporated cultural capital [26,29]. Cultural capital is not available to everyone, and like other forms of capital (e.g. monetary assets), it can serve as a currency to obtain other resources. Scarcity and exclusiveness determine its value. Individuals are usually not aware of the cultural capital they possess and put into action. Rather it takes the form of unconscious customs and beliefs and the feel for what is the right thing to do [17,24].

Cultural capital emerges in three different states: incorporated (“embodied”; e.g. dispositions and competencies), objectivised (“objectified”; e.g. possession of books, dictionaries, instruments), and institutionalised (e.g. educational qualifications) [20,26]. *Incorporated* cultural capital, “the form of long-lasting dispositions of the mind and the body”, entails socialisation, personal effort, and time investment, and becomes a part of the individual’s habitus (Bourdieu, 1986, p. 47) [26]. It is not possible to convey incorporated cultural capital to someone else, as would be possible with economic capital or objectivised cultural capital. Lareau and Weininger ([28], p. 156) refer to incorporated cultural capital as “the legitimate cultural attitudes, preferences and behaviours […] that are internalized during the socialization process”. For socioeconomic inequalities in food choice behaviour, we expect incorporated cultural capital to have the largest potential to be on the causal chain between socioeconomic position and food choices. In our study, we hypothesize to find the strongest associations of cultural capital with food choices for the incorporated state. Also Abel emphasised the importance of incorporated cultural capital for health behaviour [19,20], consistent with Bourdieu [17,26].

*Objectivised* cultural capital, i.e. books, equipment, or a work of art, can be transferred to other people, just as money can be transferred. Nevertheless, proper use of objectivised cultural capital is closely tied to incorporated cultural capital: a work of art becomes meaningful only to those who know how to evaluate its value. The third type of cultural capital is the *institutionalised* state, i.e. official (school) diplomas, ‘guarantees’ properties of incorporated capital, and was described by Bourdieu as “legally guaranteed qualifications, formally independent of the person of their bearer” ([26], p. 50).

Aside from cultural capital, Bourdieu defined two other forms of capital to describe the composition of lifestyle attributes related to social class: economic capital and social capital [18]. *Economic capital* includes all sources of income; *social capital* encompasses the “aggregate of the actual or potential social resources deriving from group membership” ([26], p. 51). Economic and social capital are linked to cultural capital by the access they provide to education and social networks (e.g. tuition and club-membership fees). The other way around, cultural capital determines accumulation and deployment of economic and social capital; for instance, education may give access to better-paid jobs, and shared norms and values are necessary to enter certain social networks [20,26].

A fundamental question underlying our search for indicators of cultural capital is: when does culture become capital, i.e. when does culture take the form of a currency that can be deployed to get ahead in life, when is it valued as giving an individual a social advantage? For cultural capital in relation to socioeconomic inequalities in food choice behaviour, the advantage would be good health. The practice or strategy of action ([30], p. 39) would be healthy food choice, and the currency or cultural capital would be the resources an individual possesses and employs to make healthy food choices. Thus, how exactly can we operationalise this cultural capital to make healthy food choices?

## Methods

### Systematic review—methods

We conducted a review of the literature in a broad range of databases, in order to get a complete overview of existing quantitative measures of cultural capital. We searched the databases PubMed, PsychInfo, Web of Science, and JSTOR with the key-word ‘cultural capital’ as a search term in ‘title’ and/or ‘abstract’ (e.g. PubMed search strategy: (cultural*[tiab] AND capital*[tiab])). Inclusion criteria for studies were: cultural capital was quantitatively measured in an empirical study, studies were published as research articles in peer-reviewed journals, in the English language, and published between 1979 (first publication year of Bourdieu’s book ‘Distinction’ [17]) and October 2010. TJ scanned the retrieved articles for the term ‘cultural capital’ in either title and/or abstract, and assessed abstracts for quantitative measurement of cultural capital; the other criteria were used as search limits in the databases. If the measurement of cultural capital could not be derived from the abstract, we labelled the article as ‘potentially relevant’ and included the article for viewing the full text. When, after reading full texts, it turned out a study did not include at least one quantitative measure of cultural capital, we excluded the article.

Articles that met all inclusion criteria were classified according to their research field. Further, we extracted indicators of cultural capital from the included articles and classified them according to the three states of cultural capital, i.e. objectivised, incorporated, and institutionalised. We distinguished between indicators measured at the individual level (i.e. cultural capital measured as activities and/or knowledge of the respondent) and family level (cultural capital measured as activities and/or knowledge of the respondent’s parents), or a combination of the two. Some studies drew from the same data source; nevertheless we evaluated all studies separately, since the applied indicators differed.

### Questionnaire development—methods

The review of existing measures of institutionalised, objectivised and incorporated cultural capital was used as main input for the development of the questionnaire to measure food choice relevant cultural capital. The most often applied indicator(s) were used for translation into measures of food choice relevant cultural capital, using existing questionnaires where possible. If a predominant measure did not come forth from the review, we used the main concepts underlying the different indicators, and searched the literature to find existing questionnaires to measure these concepts. The questionnaire was pilot-tested for readability, comprehensibility and completion time among a convenience sample of 41 adults (30% male, mean age 46 years, 49% high educated), partly consisting of friends of the second author, and partly of patients to a physiotherapy practice. Pilot participants filled in the questionnaire (including four additional questions on completion time and questions that were unclear, difficult, or unpleasant to answer) at home, and returned it to the second author directly, or on their next visit to the physiotherapy practice. Based on the results of the pilot studies, several questions were rephrased or excluded.

### Empirical study—methods

The developed questionnaire was included in a large-scale postal survey in 2011, administered in a new wave of data collection for the longitudinal cohort study called GLOBE (more information in [31–33]). Of the respondents to the previous survey in 2004 (N = 4,784) which formed a stratified sample of the 25–75 years old population in the city of Eindhoven and surrounding cities in 2004, n = 249 had died, n = 76 had emigrated, and n = 14 were lost to follow-up (i.e. no correct address information available), which resulted in a sample of N = 4,437 that was sent the 2011-survey. For the total of 2,983 respondents that returned the survey (response 67.2%), missing values for sex (n = 21), age (n = 24), and educational level (n = 172) could largely be replaced by information from the 2004-questionnaire, resulting in only one case with a missing value for age and 29 cases with missing values for educational level. These 30 respondents were excluded from the analysis, resulting in an analytic sample of N = 2,953. The use of personal data in the GLOBE study is in compliance with the Dutch Personal Data Protection Act and the Municipal Database Act, and has been registered with the Dutch Data Protection Authority (number 1248943). No formal approval of the medical ethics committee of the Erasmus University Medical Centre was required for the study.

In this 2011-survey, socioeconomic position was operationalised by the respondent’s highest attained educational level, which is considered a good indicator of socioeconomic position in the Netherlands [34] (measured with four categories, i.e. 1 = no education or primary education; 2 = lower vocational and intermediate general education; 3 = intermediate vocational and higher general education; and 4 = higher professional education and university).

With a Food Frequency Questionnaire that was part of the 2011-survey [35–37], we obtained self-reported information on the number of days per week that respondents consumed specific food items (categories: never, less than 1 day/week; 1–2 days/week; 3–4 days/week; 5–6 days/week; and every day), based on which total frequency outcomes were constructed for: consumption of unhealthy vs. healthy bread products (= summed frequency of eating white bread and croissants, minus the summed frequency of eating wholemeal bread and rye bread), unhealthy vs. healthy snacking (= summed frequency of eating candy bars, potato chips, and salted nuts, minus the summed frequency of eating gingerbread, Japanese mix, popcorn, and unsalted nuts), and consumption of unhealthy vs. healthy meat products (= summed frequency of eating beef, mutton, and pork, minus the summed frequency of eating fish, chicken, and vegetarian products like tofu). For analytic purposes, outcomes were dichotomised in: 1 = making more unhealthy choices, vs. 0 = making more healthy choices, or as many healthy as unhealthy choices.

Statistical analyses were conducted in SPSS 20.0. Internal consistency and item correlation of cultural capital measures were tested by Cronbach’s alpha. Preferably, principal component analysis was used to identify factors that represented the underlying concepts (results are available in S2 Tables Factor analyses). In case of a low Cronbach’s alpha (<0.5), or a high rate of missing values on one of the items, we calculated mean scores or sum scores to combine several items in one measure.

In logistic regression models, odds ratios with 95% confidence intervals were calculated for the food choice outcomes by socioeconomic position (indicated by the respondent’s highest attained educational level) controlled for age and sex, and by the cultural capital indicators, controlled for sex, age, and socioeconomic position. The analyses were weighted to reflect the source population (i.e. the 25–75 years of age adult population of the city of Eindhoven and surrounding cities in 2004) with respect to sex, age, and educational level.

## Results

### Systematic review—results

**Fig 1:**
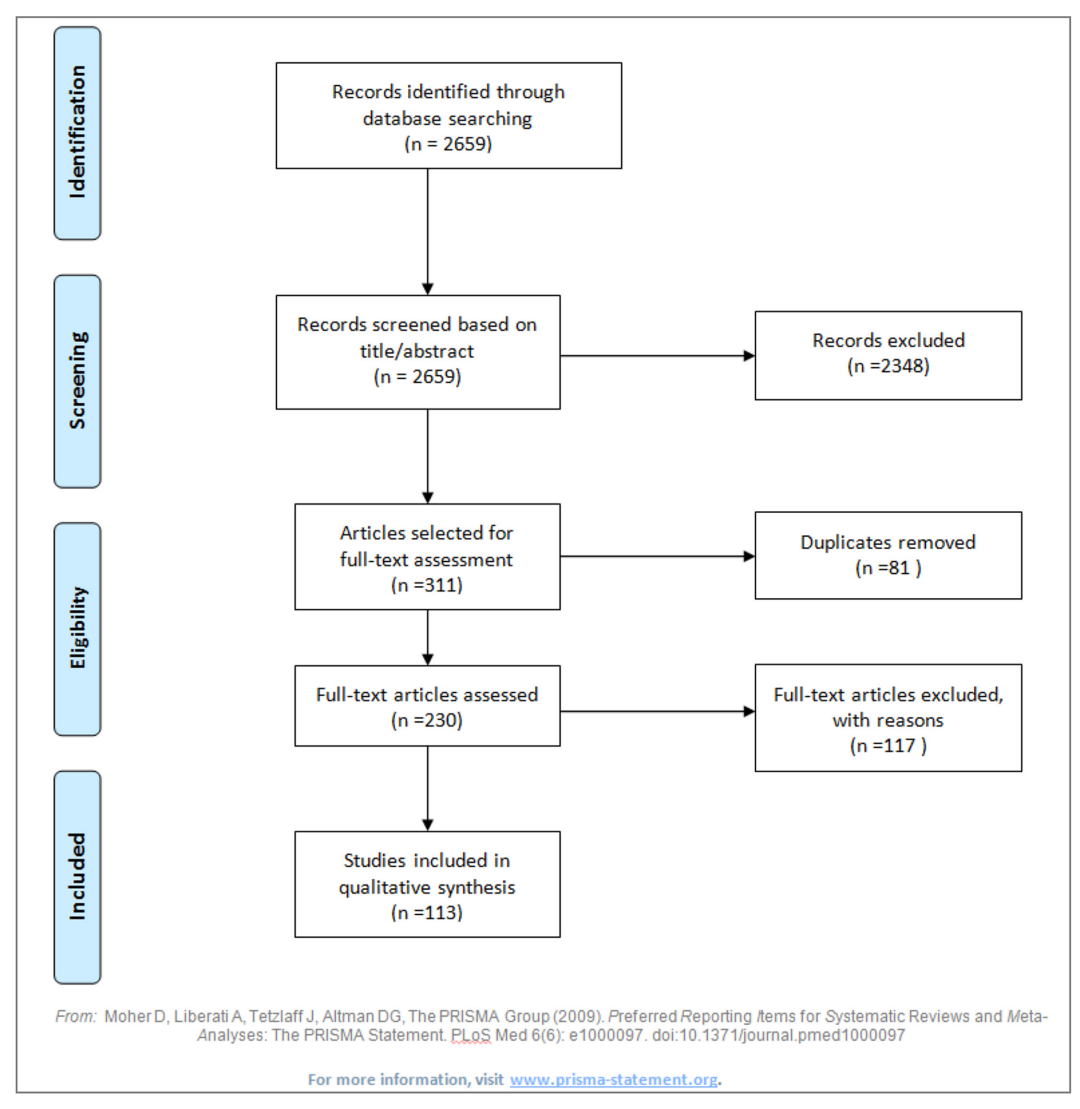
PRISMA flow diagram, regarding the inclusion of articles in the systematic review.

The literature search yielded 2,659 articles (PubMed: 48; PsychINFO 295; Web of Science: 563; JSTOR: 1,753) (see Fig 1 for the PRISMA flow chart regarding the inclusion of articles). We excluded articles when the term ‘cultural capital’ was not mentioned in the title or abstract, or when other inclusion criteria were not met. This strategy reduced the number of potentially relevant articles to 311, and to 230 after duplicates were removed. Close reading of full-texts resulted in exclusion of another 117 articles since these were either theoretical or methodological papers, reviews, or qualitative studies. Consequently, 113 articles were included in the review (see Table 1). The vast majority of studies (n = 57) was conducted in the field of educational research, mainly related to educational achievement of children (e.g. with Grade Point Average, or math and reading performance as outcome measures). Other research fields were employment/career, volunteering, parenting behaviour, and religion. Seven articles reported studies conducted in the field of (mental) health, though three ensued from the same study [25,38,39].

**Table 1:** Results of the systematic review: most prevalent indicators of cultural capital.

Indicator	Example of operationalisation	Studies in review ^a^
*Own institutionalised cultural capital*		
Education	Highest education completed by respondent	n = 15
*Family institutionalised cultural capital*		
Parental education	Highest education completed by parents	n = 19
*Objectivised cultural capital*		
Possession of cultural objects	Number of books, type of books	n = 26
	Availability of educational resources	n = 8
*Incorporated cultural capital*		
Participation	Frequency of attending cultural events, e.g. concert, cabaret, ballet, exhibitions, museums	n = 34
Skills	Acquiring skills in cultural classes, performing art	n = 17
	Reading books	n = 10
Knowledge/interest	Cultural knowledge, e.g. about literature, music, art, restaurants, sports, magazines	n = 9
	Frequency of speaking with others about books, or works of art	n = 4
Values, norms, philosophy on life	Importance of religion	n = 14

^a^ See S1 Tables Systematic review for a detailed overview of the measures found for each type of cultural capital, with references to the specific studies that included each measure

A large variety of cultural capital measures ensued from the systematic review (see S1 Tables Systematic review). *Institutionalised cultural capital* was assessed in a limited number of studies though rather consistently, namely as highest education completed (both on the individual level and family level). *Objectivised cultural capital* was consistently operationalized with indicators of ‘cultural possession’, for which five measures at the individual level and four at the family level were found, e.g. possession of books, owning works of art, and library membership. For *incorporated cultural capital*, both on the level of the individual and the family, a large variety of measures was found, with cultural participation, cultural skills, cultural knowledge, and values as main underlying themes. Attending cultural events or activities (e.g. going to concerts, visiting museums) was most often employed, with n = 34 for the individual level, and n = 19 for the family level (i.e. parents attending cultural activities and parents taking child to cultural activities together). Other often-applied operationalisations of incorporated cultural capital were cultural skills acquired through participation in classes (e.g. painting, playing a musical instrument), reading skills, cultural knowledge (e.g. of literature, music, arts), and important life values, like religion. The few studies that investigated associations of incorporated cultural capital with (mental) health outcomes also applied these types of indicators.

### Questionnaire development—results

We developed a set of questionnaire items that capture cultural capital of the three different types in relation to food choices (see Table 2). The questionnaire consisted of 56 items with an acceptable internal consistency (Cronbach’s alpha of .654).

**Table 2:** Questionnaire items to measure cultural capital related to food choices.

Indicators	Questionnaire items (56 items in total)	Answering categories	Adapted from (reference)	Variables in the analyses
*Family institutionalised cultural capital*				
Highest educational credentials of the respondent’s father, mother, and (if applicable) partner	(3 items) Please indicate the highest level of education that has been achieved by: a) your father, b) your mother, c) your partner.	1 = No education or primary education; 2 = Lower vocational education or higher general secondary education; 3 = Intermediate vocational education or higher general secondary education; 4 = higher professional education or university; Do not know; Not applicable		(1 variable)Mean score of three items, dichotomised in (1–2.51) vs. high (2.52–4).
*Objectivised cultural capital*				
Cooking equipment	(5 items) Could you please indicate whether you own the following cooking objects? a) Oven, b) Cookery book(s), c) Set of knives, d) Kitchen scales, e) Fruit juicer	Yes, no.	-	(1 variable) Sum score of five items, dichotomised in low (0–4) vs. high (5).
*Incorporated cultural capital*				
Participation	(2 items) Two items on food-related participation: a)“In the last month, how many times have you met with people in a public place to have some food?”, b) “In the last month, how many times have people visited you in your home to have dinner, or have you visited people for dinner in their home?”	Item a and b: open question.	(Grootaert et al, 2004)	(1 variable) Sum score of the two items was created, and divided in low vs. high participation.
Cooking skills	(3 items) Below you may find three statements about cooking. Please indicate for each of the following statements whether you agree or disagree. a) I know several ways to prepare fish. b) I can prepare a lot of meals even without a recipe. c) I know several ways to prepare vegetables.	Do not agree at all; Do not agree; Do not agree and do not disagree; Disagree; Disagree at all; Don’t know.	(Caraher et al., 1999; Van der Horst et al., 2011)	(1 variable) Factor analyses resulted in one factor, with scores categorised as: 1 = low cooking skills (negative scores) vs. 2 = high cooking skills (zero and positive scores)
Grocery shopping skills	(2 items) Below you may find two statements about grocery shopping. Please indicate for both statements how often this applies to you. a) Before I go shopping for food, I make a list of everything I need. b) Usually I do not decide what to buy until I am in the shop.	Always; Usually; Sometimes; Seldom; Never.	(Scholderer et al., 2004)	(1 variable) Factor analyses resulted in one factor with scores categorised as: 1 = low grocery shopping skills (negative scores) vs. 2 = high (zero and positive scores).
Food information skills	(4 items) Below are some questions about food information. Please indicate for each question how often this applies to you. A) Do you read the nutrition information and information about ingredients on food packages? b) Do you use the information about nutritional value on food packages to decide what foods you buy? c) Do you look up information about foodstuffs on the internet? (For instance on the website of the Nutrition information centre?) d) Do you use recipes from cookery books, from the internet, or from magazines?	Always; Usually; Sometimes; Seldom; Never.	(Chew et al., 2004).	(2 variables) Factor analysis of four items (item b was removed) resulted in two factors, with: 1 = low skills for use of nutrition information on food packages (negative scores) vs. 2 = high (zero and positive scores) skills, and 1 = low skills for use of nutrition information and recipes from magazines and the internet (negative scores) vs. 2 = high (zero and positive scores)
Nutrition knowledge	(16 items) Please indicate for the following four food items whether they are high or low in added sugar: a) Bananas, b) Unflavoured yoghurt, c) Ice-cream, d)Tomato ketchup. Please indicate for the following four food items whether they are high or low in protein? a) Chicken, b) Cheese, c) Fruit, d)Broccoli. Please indicate for the following four food items whether they are high or low in fibre. a) Eggs, b) Nuts, c) Chicken, d)Broccoli. Please indicate for the following four food items whether they are high or low in saturated fat? a) Olive oil, b) Nuts, c)Red meat (pork, mutton), d)Chocolate	High; Low; Don’t know	(Parmenter & Wardle, 1999; Wardle et al., 2000)	(1 variable) Sum score of all correct answers (ranging from 0–16), was divided in 1 = low (0–10) vs. 2 = high (11–16).
General human values	(21 items) Below you may find some descriptions of people. Please read each description carefully and tick the box that describes how much each person is or is not like you. Thinking up new ideas and being creative is important to him. He likes to do things in his own original way. It is important to him to be rich. He wants to have a lot of money and expensive things. He thinks it is important that every person in the world should be treated equally. He believes everyone should have equal opportunities in life.It's important to him to show his abilities. He wants people to admire what he does.It is important to him to live in secure surroundings. He avoids anything that might endanger his safety.He likes surprises and is always looking for new things to do. He thinks it is important to do lots of different things in life.He believes that people should do what they're told. He thinks people should follow rules at all times, even when no-one is watching.It is important to him to listen to people who are different from him. Even when he disagrees with them, he still wants to understand them.It is important to him to be humble and modest. He tries not to draw attention to himself.Having a good time is important to him. He likes to 'spoil' himself.It is important to him to make his own decisions about what he does. He likes to be free and not depend on others.It's very important to him to help the people around him. He wants to care for their well-being.Being very successful is important to him. He hopes people will recognise his achievements.It is important to him that the government ensures his safety against all threats. He wants the state to be strong so it can defend its citizens.He looks for adventures and likes to take risks. He wants to have an exciting life.It is important to him always to behave properly. He wants to avoid doing anything people would say is wrong.It is important to him to get respect from others. He wants people to do what he says.It is important to him to be loyal to his friends. He wants to devote himself to people close to him.He strongly believes that people should care for nature. Looking after the environment is important to him.Tradition is important to him. He tries to follow the customs handed down by his religion or his family.He seeks every chance he can to have fun. It is important to him to do things that give him pleasure.	Very much like me; Like me; Somewhat like me; A little like me; Not like me; Not like me at all.	(Schwartz, 1994), (Schwartz et al., 2001), (ESS, 2011)	(4 variables) The 21 items were computed into ten basic human values, which were aggregated to four higher-order values according to Schwartz, (i.e. Openness to change, Conservation, Self-transcendence, and Self-enhancement), and dichotomised into high = 1 and low = 0.

The review showed that institutionalised cultural capital was consistently measured by educational level, either own educational level as indicator of individual institutionalised cultural capital, or parents’ highest attained educational level as indicators of family institutionalised cultural capital. The current study is part of a broader research project into socioeconomic inequalities in food choices, in which we use the respondent’s own educational level as indicator of socioeconomic position, which therefore cannot be used as indicator of individual institutionalised cultural capital. Therefore, to measure institutionalised cultural capital, we focused on the socialisation processes in which acquisition of cultural capital takes place, and operationalised *family institutionalised cultural capital* by educational level of the father, mother and partner of the respondent (each with four categories, i.e. 1 = no education or primary education; 2 = lower vocational and intermediate general education; 3 = intermediate vocational and higher general education; and 4 = higher professional education and university). The educational level of the respondent’s father and mother may capture family cultural capital influences during childhood and adolescence to which the respondent has been exposed, and which may have influenced the respondent’s current capital levels, and current food choices. The educational level of the respondent’s partner may capture current family cultural capital influences on capital levels and food choices. The Cronbach’s alpha for these three items was .762. We calculated the mean score of these three items as overall measure of family institutionalised cultural capital. For n = 713 respondents, this mean value was based on less than three items, due to missing values. Respondents with missing values on all three items (n = 394) received a missing value for family institutionalised cultural capital. The mean score was dichotomised in 0 = low (1–2.51) vs. 1 = high (2.52–4) family institutionalised cultural capital.

*Objectivised cultural capital* was consistently measured in the literature by cultural possessions and we translated this to a list of possessions related to food choice behaviour. We asked respondents whether they owned several cooking-related possessions, i.e. stove, cook book(s), set of knives, kitchen scale, juicer, bread maker, and frying pan (yes/no) (Cronbach’s alpha: .391). The items regarding the possession of a bread maker and frying pan showed a weak correlation with the other five items. After exclusion of these two items, the Cronbach’s alpha raised to .545. A sum score was created based on the remaining five items, and divided in two categories: 0 = low (0–4) vs. 1 = high (5) objectivised cultural capital.

The measures of *incorporated cultural capital* that came forth from the literature review were wide ranging, however, we selected the main underlying themes of *participation*, *skills*, *knowledge*, and *values* as the constructs that form one’s incorporated cultural capital, and searched the literature to find existing questionnaires to measure these.

*Participation* was measured with two food-related participation items, namely (categorisation between brackets): “In the last month, how many times have you met with people in a public place to have some food?” (not at all, vs. at least once) [40], and “In the last month, how many times have people visited you in your home to have dinner, or have you visited people for dinner in their home?” (not at all, vs. at least once) [40]. A sum score of the two variables was created (ranging from 0–2), and was divided in 1 = no (0) and 2 = some (1–2) food-related participation.

For food choice related skills we distinguished three types of abilities: cooking skills [41,42], grocery shopping skills [43], and skills to find and process information about nutrients and food preparation (adapted from [44]). *Cooking skills* were measured with statements about the preparation of vegetables and fish, and the use of recipes (with answers on a 5-point Likert scale ranging from totally agree to totally not agree). Cronbach’s alpha for these three items was .773, and a factor analyses resulted in one factor that explained 70% of the variance. Scores for this factor were interpreted and categorised as: 1 = low cooking skills (negative scores) vs. 2 = high cooking skills (zero and positive scores). *Grocery shopping skills* were measured with two items on making a shopping list or deciding in store what to buy (with 5 answering options ranging from always to never). Cronbach’s alpha for these two items was .561, and a factor analyses resulted in one factor that explained 69% of the variance. Scores for this factor were interpreted and categorised as: 1 = low grocery shopping skills (negative scores) vs. 2 = high (zero and positive scores). *Food information skills* were measured with five items, for instance: “Do you read nutrition information on the labels of food products?” and “Do you use information on the labels of food products to decide what to buy?” (with 5 answering options ranging from always to never). Cronbach’s alpha for these five items was .529, and particularly the item for understanding nutrition information loaded low on all factors. After excluding this item, Cronbach’s alpha was .537, and a factor analysis on the remaining four items resulted in two factors that together explained 69% of the variance. Scores for these factors were interpreted as follows: 1 = low skills for use of nutrition information on food packages (negative scores) vs. 2 = high (zero and positive scores) skills, and 1 = low skills for use of nutrition information and recipes from magazines and the internet (negative scores) vs. 2 = high (zero and positive scores).

*Nutrition knowledge* was measured with an existing questionnaire including 16 items (Cronbach’s alpha: .519), namely four different questions (e.g. do these products contain high or low levels of added sugar? do these products contain high or low levels of protein?), that were asked with regard to four products each (e.g. bananas, chicken, chocolate, red meat) (three answer categories: high, low, don’t know) [45,46]. A sum score of all correct answers was made (ranging from 0–16), and divided in 1 = low (0–10) and 2 = high (11–16) nutrition knowledge.

We created a mean score based on the participation, skills and knowledge variables as measure of *total incorporated cultural capital*, and divided the mean score into 0 = low (1.00–1.49), vs. 1 = high (1.50–2.00).

Instead of food values-which would be too close to the taste of food, and therefore to the outcome of interest- ten distinctive general human values were constructed [47,48] by means of the Portrait Values Questionnaire of Schwartz (also applied in previous studies on food choices [49,50]), including 21 items (Cronbach’s alpha: 0.801). Values were computed excluding respondents who had more than 5 missing values or 16 similar answers on the value items, according to Bilsky, Janik, and Schwartz (2011) [51]. The ten general values were aggregated to four higher-order values [52], i.e. *openness to change* (i.e. pursuing whatever intellectual or emotional directions one wishes, however unpredictable or uncertain the outcomes), *conservation* (i.e. preserving the status quo and the certainty it provides in relationships with close others, institutions, and traditions), *self-transcendence* (i.e. transcending one's selfish concerns and promoting the welfare of others and of nature), and *self-enhancement* (i.e. enhancing one's own personal interests, even at the expense of others) and dichotomised into high = 1 and low = 0. Although considered part of one’s incorporated cultural capital, these general values were too distinct in nature from the other variables to capture in the overall sum score for incorporated cultural capital, and therefore, were analysed separately.

A variable for *total cultural capital* (excluding the general values) was created by computing the mean score of the family institutionalised, objectivised, and total incorporated cultural capital variables, which was dichotomised into low total cultural capital (mean score of 0–0.50) vs. high (mean score of 0.51–1.00). A factor analysis of all cultural capital items combined (leaving out the general values, and with a sum score for food knowledge) showed that the measures of family institutionalised cultural capital loaded on one factor with the items for nutrition information skills, and defined separate scales from those of objectivised cultural capital, cooking skills, shopping skills, and participation (results available in S2 Tables Factor analyses).

### Empirical study–results

Mean age of the sample was 56.4 years (SD 13.0) and 56.7% was female. As reported in Table 3, lower socioeconomic groups possessed significantly less cultural capital of all three types than higher socioeconomic groups. For instance, where 63.6% of the high socioeconomic group possessed a high level of incorporated cultural capital, this was 32.8% among the low socioeconomic groups. The proportion of missing values on the variables for family institutionalised and objectivised cultural capital was higher among low than high socioeconomic groups.

**Table 3:** Odds ratios for unhealthy food choices by socioeconomic position^a^ (adjusted for age and sex) and by cultural capital (adjusted for age, sex, and socioeconomic position), and prevalence rates of family institutionalised, objectivised and incorporated cultural capital by socioeconomic position^a^.

	N^b^ (%)^c^ (n = 2953)	OR for eating more unhealthy than healthy bread products (n = 2781)^d^			OR for eating more unhealthy than healthy meat products (n = 2782) ^d^			OR for eating more unhealthy than healthy snacks(n = 2736) ^d^			Socioeconomic position^a^ (%) (n = 2953) ^d^				
		OR^e^	(95% CI)	p	OR^e^	(95% CI)	p	OR^e^	(95% CI)	p	1(low)	2	3	4 (high)	*P*
Total sample	2953 (100%)														
Socioeconomic position				< .0001			< .0001			.052					
1 Low	263 (6.3%)	3.22	(1.77–5.84)		1.96	(1.37–2.80)		0.95	(0.49–1.86)		-	-	-	-	
2	1041 (31.4%)	1.56	(1.03–2.37)		1.38	(1.13–1.69)		1.52	(1.11–2.07)		-	-	-	-	
3	678 (26.5%)	2.03	(1.40–2.94)		1.60	(1.31–1.95)		1.25	(0.93–1.68)		-	-	-	-	
4 High	971 (35.9%)	1.00			1.00			1.00			-	-	-	-	
Total cultural capital				< .0001			< .0001			.004					< .0001
Low	1583 (49.8%)	2.14	(1.16–3.95)		1.43	(1.21–1.69)		1.47	(1.13–1.90)		85.5	64.8	49.1	30.8	
High	136 (50.1%)	1.00			1.00			1.00			14.0	35.1	50.9	69.1	
Missing	4 (0.1%)	-			-			-			0.5	0.1	0.0	0.1	
Family institutionalisedcultural capital				< .0001			.313			.726					< .0001
Low	1500 (49.8%)	2.33	(1.60–3.37)		1.12	(0.93–1.34)		1.04	(0.79–1.37)		54.1	68.0	51.0	32.1	
High	1059 (40.0%)	1.00			1.00			1.00			10.3	16.8	42.1	63.9	
Missing	394 (10.2%)	2.34	(1.29–4.25)		0.94	(0.69–1.28)		0.84	(0.49–1.45)		35.7	15.1	6.9	4.0	
Objectivised cultural capital				< .0001			.009			.027					< .0001
Low	1306 (42.8%)	1.87	(1.38–2.54)		1.26	(1.07–1.48)		1.24	(0.97–1. 58)		69.9	52.7	45.8	46.5	
High	1598 (56.0%)	1.00			1.00			1.00			24.2	45.9	53.5	52.9	
Missing	49 (1.3%)	2.66	(0.84–8.47)		1.87	(0.82–4.28)		2.99	(1.16–7.73)		5.9	1.4	0.8	0.6	
Incorporated cultural capital				< .001			< .0001			< .0001					< .0001
Low	1420 (46.0%)	1.65	(1.21–2.24)		1.72	(1.47–2.02)		1.73	(1.34–2.21)		66.7	54.2	45.4	36.1	
High	1525 (53.8%)	1.00			1.00			1.00			32.8	45.6	54.6	63.6	
Missing	8 (0.2%)	-			-			-			0.5	0.2	0.0	0.3	
General values															
Openness to change				.005			.023			0.80					< .0001
Low	905 (29.9%)	1.66	(1.22–2.27)		1.27	(1.07–1.51)		1.09	(0.83–1.42)		38.4	35.1	29.5	24.1	
High	1992 (68.6%)	1.00			1.00			1.00			57.8	62.8	69.9	74.5	
Missing	56 (1.6%)	1.73	(0.49–6.06)		1.02	(0.50–2.12)		1.18	(0.39–3.54)		3.8	2.2	0.6	1.4	
Conservation				.257			.228			.403					< .0001
Low	647 (23.9%)	1.22	(0.87–1.71)		0.86	(0.71–1.04)		1.20	(0.92–1.58)		15.6	16.8	25.8	30.2	
High	2249 (74.4%)	1.00			1.00			1.00			79.6	80.9	73.6	68.4	
Missing	57 (1.7%)	1.97	(0.69–5.64)		0.74	(0.35–1.53)		1.12	(0.37–3.32)		4.8	2.3	0.6	1.4	
Self-transcendence				.013			.808			.085					< .0001
Low	110 (3.5%)	1.90	(1.03–3.52)		0.87	(0.56–1.33)		1.83	(1.07–3.13)		9.1	4.3	3.1	2.1	
High	2787 (94.9)	1.00			1.00			1.00			87.6	93.3	96.3	96.5	
Missing	56 (1.6%)	2.92	(1.12–7.66)		0.99	(0.48–2.04)		1.16	(0.39–3.45)		3.2	2.4	0.6	1.4	
Self-enhancement				.843			.727			.067					< .0001
Low	2020 (66.4%)	1.02	(0.74–1.41)		0.93	(0.79–1.11)		0.74	(0.58–0.96)		75.8	72.4	67.5	58.8	
High	875 (31.9%)	1.00			1.00			1.00			20.4	25.4	31.8	39.8	
Missing	58 (1.6%)	1.46	(0.41–5.16)		0.89	(0.43–1.85)		0.95	(0.31–2.86)		3.8	2.3	0.6	1.4	

^a^ Socioeconomic position was measured by the respondent’s highest attained educational level.

^b^ The numbers (N) are unweighted and reflect the actual numbers of participants in the dataset.

^c^ The percentages (%) are weighted and thereby represent the prevalence rates as they existed in the population of Eindhoven of 2004, which is the source population. The weight factors were calculated from the distribution of the characteristics in a random sample drawn from the municipal registry in Eindhoven, October 2004.

^d^ Varying sample sizes due to exclusion of missing values on the specific food choice outcome.

^e^ Odds ratios (OR) for unhealthy food choices by socioeconomic position were adjusted for sex and age. Odds ratios for unhealthy food choices by cultural capital variables and general values were adjusted for sex, age and socioeconomic position.

Low socioeconomic groups were more likely to eat more unhealthy than healthy bread products (OR 3.22 (95% CI: 1.77–5.84)), and to eat more unhealthy than healthy meat products (OR 1.96 (95% CI: 1.37–2.80)) than higher socioeconomic groups (controlled for age and sex). The second-lowest group was more likely to consume more unhealthy than healthy snacks (OR 1.52 (95% CI: 1.11–2.07) compared to the highest socioeconomic group.

Controlled for age, sex, and socioeconomic position, low levels of total cultural capital were associated with unhealthy food choices. Of the three subtypes of cultural capital, the incorporated state showed the most consistent associations with unhealthy food choices, and in the expected direction: low levels of incorporated cultural capital were associated with more unhealthy food choices. Bread consumption was associated with all cultural capital variables, whereas meat consumption and snacking were only significantly associated with total and incorporated cultural capital. Regarding the general values, low educated were less open to change and more conservative than high educated, and had lower scores for self-transcendence and self-enhancement. A low openness to change and low self-transcendence seemed to be associated with making less healthy food choices, especially for bread consumption.

## Discussion

Our systematic review showed that the concept of cultural capital has not yet been empirically tested in relation to healthy and unhealthy food choices. Several indicators of institutionalised (e.g. educational level) and objectivised cultural capital (e.g. possession of books, art) have consistently been applied in other research fields, whereas incorporated cultural capital has been measured with a large variety of indicators (e.g. cultural participation, skills). During questionnaire development, indicators of institutionalised and objectivised cultural capital were translated to food choice relevant indicators. For incorporated cultural capital, we used existing questionnaires that measured the larger concepts that covered the variety of indicators as identified by the review (i.e. participation, skills, knowledge, values). An empirical test of the cultural capital questionnaire showed an acceptable overall internal consistency (Cronbach’s alpha of .654; 56 items). Higher socioeconomic groups significantly retained more cultural capital of all three states than those from lower socioeconomic groups, and having more cultural capital was associated with healthier food choices.

The main strength of the present study is our comprehensive approach for the development of a set of measures for food choice relevant cultural capital. The literature review resulted in an inclusive overview of all cultural capital indicators and measures that have been applied in different fields of research, from the early conceptualisations to recent understandings of the concept of cultural capital. We were thereby more inclusive than a previous review of selected studies that applied cultural capital in educational research [28]. Since we restricted the search term to ‘cultural capital’ we may have missed studies that included similar measures but did not label them as cultural capital. A limitation of the cultural capital questionnaire is that it is mostly food-specific; only the questions regarding family institutionalised cultural capital and the general values may also be applicable to other health-behaviours, like physical activity. However, the advantage of this approach is that the food-choice specific cultural capital variables are more likely to have causal associations with food choices than general cultural capital indicators (like museum visits, and possession of art), which make them more useful as entry points for intervention development to promote healthy food choices. Another limitation may be that the selected questionnaire items are especially applicable to Western cultures similar to the Dutch culture; for application in other cultures, some questionnaire items may need some modification (e.g. regarding nutrition knowledge, and possession of cooking equipment). A limitation of the empirical test is that food intake was based on self-reports, with social desirability bias as a potential problem.

We combined the results of the literature review with existing questionnaires on the underlying concepts of the three states of cultural capital. Especially with regard to incorporated cultural capital, clearly a gap exists between Bourdieu’s abstract ideas and the operationalisation into measurable constructs of incorporated cultural capital, as the measures of incorporated cultural capital that came forth from the literature review were wide ranging. Therefore, we selected the main underlying concepts of participation, skills knowledge, and values-which are also prominent in the original writings of Bourdieu [17,26] and others [20,28]. Measures for skills and knowledge that were selected from the food choice literature sometimes needed some adaption to the Dutch situation, as, for instance, the questions on food knowledge included particular British food items not well known in the Netherlands.

We followed Abel [20] by appreciating values as reflecting incorporated cultural capital, since values are typically ‘long lasting dispositions of the mind’ [26], and guide what individuals appraise as important in their lives [53–55]. Values are both individual characteristics and cultural patterns of social class [56] and have a major influence on lifestyle and consumption patterns [57]. We measured general values instead of food-choice related values since the food values would be too close to the taste of food, and therefore to the outcome of interest itself. However, the general values were rather distinctive in nature from the other food-choice specific incorporated cultural capital items so that these could not be summed in the overall food-choice related incorporated cultural capital sum score.

In additional univariate analyses, we separately investigated the association of each specific variable (that was used to compose the cultural capital variables) with food choices, adjusted for sex, age and socioeconomic position (see S3 Tables Cultural capital and food choices). Results indicated that the educational level of the respondent’s father, mother and partner had strong positive associations with bread choices, while for the other two outcomes associations were weaker. The possession of a juicer (one of the items used as indicator of objectivised cultural capital) had consistent significant associations with all three food choice outcomes. Skills for preparing vegetables and fish, reading nutrition information on food packages, and food knowledge (which were all used as indicators of incorporated cultural capital) also showed consistent positive associations with healthy food choices. The two food participation indicators were positively related with healthier meat choices, but less strongly with the other two food choice outcomes.

The review showed that own educational level was the most often used indicator of *institutionalised cultural capital* at the individual level. However, as own educational level was used as indicator of socioeconomic position in our study, and as we were looking for factors that can explain socioeconomic inequalities in food choices, we focused on the socialisation processes in which acquisition of cultural capital takes place, and operationalised family institutionalised cultural capital by educational level of the father, mother and partner of the respondent. What the best way is to operationalise institutionalised cultural capital and socioeconomic position when both are needed from a conceptual point of view deserves attention in future studies.

For most cultural capital items, the proportion of missing values was acceptable, ranging between 1–5%. Only for the family institutionalised cultural capital items, a large number of respondents missed data on educational level of their partner (19.1%), father (26.7%) and/or mother (26.1%), with the largest proportion of missing values among respondents with a low socioeconomic position. In further analyses, we will apply multiple imputation on the separate cultural capital items, before combining them into mean scores or sum scores.

We were conscientious in our attempt to develop new indicators of cultural capital that did justice to Bourdieu’s ideas. Empirical assessment of the cultural capital questionnaire suggested promising associations with socioeconomic position and several food choices, and will soon be explored further for other food choice outcomes. Also, we will further explore associations of food-related cultural capital with Bourdieu’s general indicators of cultural capital (e.g. possession of books and works of art), and associations of cultural capital with social and economic capital in relation to inequalities in food choices. Also, future research may study cultural capital in relation to other health behaviours, such as physical activity. Questionnaire items regarding objectivised and incorporated cultural capital should then be adjusted accordingly.

### Conclusions

This study’s systematic review identified a large variety of cultural capital indicators which served as main input for the development of a cultural capital questionnaire. An empirical application of the questionnaire demonstrated that higher educated people significantly possessed more cultural capital, and those with a high level of cultural capital more often made healthier food choices as opposed to those with low cultural capital. Cultural capital may be a new, potentially powerful explanation for inequalities in food choices, which may lead to new entry points for the development of interventions to promote healthy food choices among low socioeconomic groups.

## Supporting Information

S1 Checklist Prisma 2009. (DOC)

S1 Dataset (XLS)

S1 Tables Systematic review. (DOC)

S2 Tables Factor analyses. (DOC)

S3 Tables Cultural capital and food choices. (DOC)
