# Supplementary material for: Bourdieu’s Cultural Capital in Relation to Food Choices: A Systematic Review of Cultural Capital Indicators and an Empirical Proof of Concept
Source: PLoS One. 2015 Aug 5;10(8):e0130695. doi: 10.1371/journal.pone.0130695 (PMC4526463; doi:10.1371/journal.pone.0130695)
Supplement: S1 Tables — (DOC) [file pone.0130695.s003.doc]

# Web-appendix 1: Tables with results of the systematic literature review

**Table 1 References of articles included in review**

|  | **Reference** | **Source** |  |  | **Reference** | **Source** |
| --- | --- | --- | --- | --- | --- | --- |
| 1  2  3  4  5  6  7  8  9  10  11  12  13  14  15  16  17  18  19  20  21  22  23  24  25  26  27  28  29  30  31  32  33  34  35  36  37  38  39  40  41  42  43  44  45  46  47  48  49  50  51  52  53 | (Adamuti-Trache and Andres 2008)  (Aschaffenburg and Maas 1997)  (Barone 2006)  (Corten and Dronkers 2006)  (De Graaf and De Graaf 2002)  (De Graaf et al. 2000)  (De Graaf 1986)  (DiMaggio 1982)  (DiMaggio and Mohr 1985)  (Driessen and Smit 2007)  (Dumais 2002)  (Dumais 2006a)  (Dumais 2006b)  (Dumais and Ward 2010)  (Eitle and Eitle 2002)  (Flere et al. 2010)  (Georg 2004)  (Jæger 2009)  (Jonsson 1987)  (Kalmijn and Kraaykamp 1996)  (Karen 1991)  (Katsillis and Rubinson 1990)  (Kaufman and Gabler 2004)  (Kingston et al. 2003)  (Lamb 1989)  (Lampard 2007)  (Lee and Kao 2009)  (Mickelson and Greene 2006)  (Myrberg and Rosen 2006)  (Myrberg and Rosen 2008)  (Myrberg and Rosen 2009)  (Noble and Davies 2009)  (Nora 2004)  (Oates 2009)  (Orr 2003)  (Pearce and Lin 2007)  (Perreira et al. 2006)  (Powell et al. 2006)  (Roscigno and Ainsworth-Darnell 1999)  (Roscigno et al. 2006)  (Salisbury et al. 2009)  (Scherger and Savage 2010)  (Solhaug 2009)  (Strayhorn 2010)  (Sullivan 2001)  (Tramonte and Willms 2010)  (Turmo 2004)  (Van de Werfhorst et al. 2003)  (Van de Werfhorst and Hofstede 2007)  (Van de Werfhorst and Kraaykamp 2001)  (Van der Velden 1996)  (Vryonides 2007)  (Wildhagen 2009) | IJSE  ASR  Socio.  ERE  NJSS  SE  SE  ASR  AJS  AS  SE  Poetics  SocS  Poetics  SE  BJSE  ESR  SForc.  ESR  SE  SForu.  ASR  Poetics  SE  SE  SRO  Poetics  JNE  JER  ERE  JEP  BJSE  JHHE  SPE  SE  ER  Demo.  SForc.  SE  SForc.  RHE  SR  JCAL  RHE  Socio.  EE  SJER  BJS  SE  BERJ  NJSS  BERJ  SQ |  | 54  55  56  57  58  59  60  61  62  63  64  65  66  67  68  69  70  71  72  73  74  75  76  77  78  79  80  81  82  83  84  85  86  87  88  89  90  91  92  93  94  95  96  97  98  99  100  101  102  103  104  105  106  107 | (Wu 2008)  (Yamamoto and Brinton 2010)  (Zimdars et al. 2009)  (Zarycki 2007)  (Erickson 1991)  (Erickson 1996)  (Fujimoto 2004)  (Johnson et al. 1995)  (Johnson et al. 2000)  (Kay and Hagan 1998)  (Kim et al. 2006)  (Osinsky and Mueller 2004)  (Pinheiro and Dowd 2009)  (Egerton 1997)  (Choi et al. 2007)  (Choi and Chou 2010)  (Kim et al. 2007)  (Okun and Michel 2006)  (Parboteeah et al. 2004)  (Tang 2006)  (Wilson and Musick 1997)  (Kanaan and Afifi 2010)  (Khawaja et al. 2007).  (Khawaja and Mowafi 2006)  (Khawaja and Mowafi 2007)  (Kim and Kim 2009)  (Tonmyr et al. 2006)  (Achterberg and Houtman 2006)  (Becker 2010)  (Böröcz and Southworth 1996)  (DiMaggio and Mukhtar 2004)  (Jagodzinski 2010)  (Katz-Gerro and Sullivan 2010)  (Lee 2009)  (Lewicka 2005)  (Lizardo 2005)  (Lizardo 2006)  (Marjoribanks and Kwok 1998)  (Mehus 2005)  (Mitchell 1994)  (Pedersen 1996)  (Pellerin and Stearns 2001)  (Pettit 1999)  (Prieur et al. 2008)  (Robinson and Garnier 1985)  (Roose and Vandenhaute 2010)  (Stempel 2005)  (Tanner et al. 2008)  (Trienekens 2002)  (Van Wel et al. 2006)  Wang, Davis, and Bian  (Wuthnow 2000)  (Adoni 1995)  (Houtman 2000) | SP  SE  Socio.  RES  CRS  AJS  SQ  RBPEUAR  ASR  SBE  WO  Poetics  WES  RA  AS  JG  JAG  JWB  JAG  ASR  PHN  Child.  JUH  JPH  SIR  IJMHP  EJPR  ESR  SForc.  Poetics  SIR  TS  CAN  JEP  Poetics  ASR  PR  IRSS  SP  AS  Poetics  Poetics  Poetics  AJS  Poetics  IRSS  BJS  Poetics  Poetics  MC  JSSR  JC  NJSS |

**Table 2 Research field of the studies included in review**

| **Resarch field** |  | **Example research question / objective** |
| --- | --- | --- |
| Education  (1-57) | (n=57) | What are the effects of parental cultural capital and own cultural capital acquired both in and outside of school on the likelihood of making educational transitions? |
|  |  | Do academic rewards in the Greek educational system reflect family background inequalities? |
|  |  | Study the influence of four forms of capital (human, cultural, school, and community capital) on high school dropout rates by immigrant generation and racial-ethnic groups. |
| Employment/career  (58-67) | (n=10) | What part does culture play in concrete class structure in work organisations? |
| Volunteering  (68-74) | (n=7) | Examine whether human, cultural, and social capital associated with volunteering among elders in the United States is equally important in Korea. |
| Health  (75-80) | (n=6) | Examine association between cultural capital and self-rated psychosocial health among poor ever-married Lebanese women living in urban context. |
| Other  (81-107) | (n=27) | Study the relationship between ethnic prejudice and social class of origin, cultural capital, own education/work, and gender. |
|  |  | Investigate relationship between cultural capital and different forms of sport consumption |

**Table 3 Objectivised Cultural Capital**

| **Indicator** | **Measure** |  | **Reference** |
| --- | --- | --- | --- |
| *Individual* | | | |
| Cultural possession | Number of books one owns | (n=6) | (17, 34, 81, 83, 88, 107) |
|  | Type of books one owns (e.g. poetry, classic literature) | (n=2) | (3-4) |
|  | Owning works of art (e.g. paintings) | (n=2) | (3-4) |
|  | Owning educational objects (e.g. computer, dictionary, internet access, encyclopaedia) | (n=2) | (34, 52) |
|  | Availability of a musical instrument in the home | (n=2) | (35, 46) |
|  | Membership of public library | (n=1) | (32) |
| *Family* | | | |
| Parental cultural possession | Number of books parents own (also children’s books) | (n=15) | (15, 30-31, 39, 43, 45-47, 49, 54-57, 88, 94) |
|  | Availability of educational resources in the home (e.g. dictionary, desk to study, quiet place to study, computer) | (n=6) | (15, 18, 39, 47, 54-55) |
|  | Subscription to daily newspaper and/or magazine | (n=4) | (34, 39, 45, 51) |
|  | Type of books parents own (e.g. poetry, classic literature) | (n=3) | (18, 32, 46) |
|  | Works of art parents own (e.g. paintings) | (n=3) | (18, 46, 55) |

**Table 4 Incorporated Cultural Capital at the Individual level**

| **Indicator** | **Measure** |  | **Reference** |
| --- | --- | --- | --- |
| Participation | Frequency of attending cultural events (e.g. theatre, concert, cabaret, ballet, exhibitions, museums, cinema) | (n=34) | (14-17, 22-25, 27, 32, 34, 38-40, 45-47, 52-53, 56, 58-59, 75, 79, 81, 83-84, 89-90, 96, 101-103, 107) |
|  | Watching television (e.g. drama, comedy, news programmes), listening to radio | (=6) | (32, 45, 77-78, 89, 94) |
|  | Participation in sports (e.g. tennis, soccer, swimming) | (n=3) | (23, 27) |
|  | Participation in school clubs (e.g. cheerleading, philosophy club, newspaper club) | (n=3) | (23, 27)  (44* Cultural and social capital together) |
|  | Frequency with which one spoke with others about arts, culture, books | (n=4) | (17, 46, 81, 107) |
|  | Listening to classical music | (n=3) | (17, 32, 55) |
|  | Volunteering for cultural organisation | (n=2) | (77-78) |
|  | Involvement in hobbies | (n=1) | (101) |
|  | Number of trips taken abroad | (n=1) | (83) |
|  | Eating out (e.g. type of restaurants) | (n=1) | (58) |
|  | Level of involvement that might expose student to networks, resources, attitudes, values (e.g. study with friends, participate in extra-curricular activities, use a computer for homework) | (n=1) | (41* Cultural and social capital together) |
| Cultural skills | Participation in cultural classes (e.g. music, visual arts, performance, non-English language instruction)    Context where cultural classes were attended (e.g. at school, outside  school) | (n=15)  (n=1) | (2, 11-13, 15, 23, 28, 34-35, 38-40, 53, 87, 103)  (2) |
|  | Extent to which one performs art (e.g. creating visual arts, performed publicly, playing musical instrument, writing texts) | (n=9) | (8-9, 16-17, 77-78, 96, 101, 103) |
| Reading skills | Frequency reading books | (n=10) | (16-17, 32, 52, 56, 81, 83, 101, 104, 107) |
|  | Type of books read | (n=6) | (32, 58-59, 89-90, 104) |
|  | Type of reading (books, newspapers, poetry, plays) | (n=3) | (77-78, 89) |
|  | Type of newspaper read | (n=2) | (32, 86) |
|  | Attending classes for advanced reading | (n=1) | (17) |
|  | Reading abilities (e.g. children’s early reading, letter knowledge) | (n=1) | (31) |
| Cultural knowledge | Cultural knowledge (e.g. about literature, music, art, restaurants, sports, magazines) | (n=8) | (8-9, 45, 56, 58-59, 96, 104) |
|  | Children’s knowledge about famous places (e.g. Leaning tower of Pisa), and famous characters (e.g. Mickey Mouse) | (n=1) | (82) |
| Attitude | Extent to which one regards oneself a lover of arts and culture (e.g. enjoying beautiful things, being a cultured person) | (n=6) | (8-9, 81, 95-96, 107) |
|  | Interest in artistic activities (e.g. artistic, musical, literary, writing) | (n=4) | (8-9, 24)  (41* Cultural and social capital together) |
|  | Interest / attractiveness careers | (n=3) | (8-9, 97) |
|  | Educational aspirations (desire to go to college) | (n=2) | (37) (41* Cultural and social capital together) |
|  | School attachment (e.g. feeling close to people at school, feeling part of the school) | (n=1) | (37) |
| Religion | Perceived importance of religion | (n=6) | (68-69, 71, 73-75) |
|  | Participation in religious services | (n=5) | (61, 71, 73-74, 85) |
|  | Religious identity (consider oneself as religious) | (n=3) | (69, 71, 75) |
|  | Ethnic religious background (e.g. white Anglo-protestant) | (n=1) | (63) |
|  | Type of religion (e.g. Buddhism, Catholicism) | (n=1) | (70) |
| Feminine cultural capital | Dependency on the family (e.g. commuting from family’s home vs. student’s home to university, ability to manage household tasks) | (n=1) | (60) |
| Working status | Professional experience (e.g. years in the profession) | (n=1) | (65) |
|  | Work status (US citizens or alien) | (n=1) | (62) |
| Generative concern | Statements (e.g. Others would say that you have made unique contributions to society, you like to teach things to people) | (n=1) | (71) |
|  | Statement (life is not worth living if one cannot contribute to the well-being of other people) | (n=1) | (74) |
| Language | Speaking foreign language(s) | (n=1) | (83) |
|  | Language (active and passive vocabulary test scores) | (n=1) | (45, 61) |
| School performance | Performance in college (e.g. college GPA) | (n=1) | (65) |

**Table 5 Incorporated Cultural Capital at the Family level**

| **Indicator** | **Measure** |  | **Reference** |
| --- | --- | --- | --- |
| Parental participation | Frequency parents attending cultural activities (e.g. theatre, concert, cabaret, ballet, exhibitions, museums) | (n=12) | (5-7, 10-11, 14, 20, 32, 45, 48, 76, 103) |
|  | Parents taking respondent/child to cultural events during childhood (e.g. art museums, galleries, plays, dance, classical musical performance) | (n=7) | (2, 11-12, 35, 42, 55, 91) |
|  | Frequency parents watch television programmes (e.g. drama, comedy, news programmes), listening to radio | (n=4) | (32, 45, 76, 94) |
|  | Listening to music with parents | (n=3) | (3-4, 46) |
|  | Frequency parents visit library (borrowing books) | (n=3) | (7, 11, 23) |
|  | Parental cultural activities at home (e.g. listening to classical music, opera) | (n=2) | (2, 23) |
|  | Maternal volunteering for cultural organisation | (n=1) | (76) |
|  | Discussing with parents about politics, social issues, books, films, television programmes | (n=5) | (3-4, 18, 46, 105) |
|  | Parents discuss books, art, science, current affairs, music (with each other) | (n=2) | (32, 45) |
|  | Mother’s religious participation (attending religious services) | (n=1) | (87) |
|  | Society religious participation (percentage of people attending religious services weekly) | (n=1) | (72) |
| Parental cultural skills | Maternal participation in art production (e.g. playing musical instrument, making art objects) | (n=2) | (76, 103) |
| Parental reading skills | Frequency parents read books | (n=7) | (6-7, 45, 50-51, 54, 106) |
|  | Type of books parents read (e.g. thrillers, translated literature, literature in foreign language) | (n=7) | (5-6, 32, 48-50, 54) |
|  | Reading/telling stories to child | (n=5) | (30-31, 50-51, 55) |
|  | Newspaper reading (type of newspaper) | (n=4) | (32, 48, 50, 54) |
| Strategic interaction / encouragement / involvement | Parental involvement (e.g. discuss school activities with parents, parents spoke with teacher or counsellor) | (n=4) | (18, 36, 46, 51) |
|  | Parental encouragement to read (not for school), and/or to write stories, play an instrument, taking part in sport, etc. | (n=3) | (2, 20, 42) |
|  | Parenting style (e.g. limiting watching television, parents trust their child to do what they expect, monitoring child’s friendships) | (n=2) | (36-37) |
|  | Receiving help from others with college application (e.g., filling out forms, writing essay) | (n=1) | (14) |
|  | Parent – school interaction (e.g. contacting school about post-high school educational opportunities) | (n=1) | (14) |
|  | Family encouragement in musical involvement (e.g. attention, financial attention) | (n=1) | (66) |
|  | Cultural pedagogic level, i.e. parents stimulate cognitive development of their children (e.g. correcting word use, teaching songs) | (n=1) | (51) |
|  | Discussion with parents about college | (n=1) | (44* Cultural and social capital together) |
|  | Parental closeness (e.g. satisfaction, warmth in parent-child relationship) | (n=1) | (37) |
|  | Family encouragement attending college (e.g. my decision to attend this university was based on support offered by my family) | (n=1) | (33) |
|  | Institutional support (e.g. my decision to attend this university was based on support offered by an academic advisor) | (n=1) | (33) |
| Parental working  status | Occupation | (n=3) | (21, 80, 93) |
|  | Father’s employment status (e.g. owning business with employees, without employees, being manager, being employee) | (n=1) | (63) |
|  | Mother’s labour participation (e.g. fulltime, part-time, unpaid) | (n=1) | (63) |
|  | Whether parents are business owners | (n=1) | (64) |
| Background | Family background influences (e.g. living with both parents until 16^th^ birthday, receiving welfare in family) | (n=2) | (61-62) |
|  | Race/ ethnic identity (e.g. white, African American, Hispanic origin) | (n=1) | (61) |
|  | Geographic influences (e.g. where mother was living when individual was born) | (n=1) | (61) |
|  | Mother’s attachment to racial/ethnic heritage | (n=1) | (87) |
|  | Political influences (e.g. republican, democrat, registration to vote) | (n=1) | (61) |
|  | Third world socialisation (e.g., born in Third world country, receiving education mostly outside US) | (n=1) | (62) |
|  | Southern roots (e.g., born and lived primarily in that region prior to age 16) | (n=1) | (62) |
|  | Ever lived in public housing | (n=1) | (62) |

**Table 6 Institutionalised Cultural Capital**

| **Indicator** | **Measure** |  | **Reference** |
| --- | --- | --- | --- |
| *Individual* |  |  |  |
| Education | Education completed | (n=15) | (17, 26, 60, 63, 65, 81, 83, 88, 92, 97-100, 106-107) |
|  | Educational tracking | (n=2) | (60, 99) |
| *Family* |  |  |  |
| Parental education | Father’s and/or mother’s completed education | (n=19) | (1, 16-17, 19, 21, 26, 29-31, 41, 44, 47-48, 57, 67, 80, 88, 97-98) |
|  | Highest educational expectation | (n=2) | (36, 44 * Cultural and social capital together) |

# **References**

Achterberg P, Houtman D (2006) Why do so many people vote 'unnaturally'? A cultural explanation for voting behaviour. European Journal of Political Research 45(1):75-92

Adamuti-Trache M, Andres L (2008) Embarking on and persisting in scientific fields of study: Cultural capital, gender, and curriculum along the science pipeline. International Journal of Science Education 30(12):1557-1584 doi:10.1080/09500690701324208

Adoni H (1995) Literacy and reading in a multimedia environment. Journal of Communication 45(2):152-174

Aschaffenburg K, Maas I (1997) Cultural and educational careers: The dynamics of social reproduction. American Sociological Review 62(4):573-587

Barone C (2006) Cultural capital, ambition and the explanation of inequalities in learning outcomes: A comparative analysis. Sociology-the Journal of the British Sociological Association 40(6):1039-1058 doi:10.1177/0038038506069843

Becker B (2010) The Transfer of Cultural Knowledge in the Early Childhood: Social and Ethnic Disparities and the Mediating Role of Familial Activities. European Sociological Review 26(1):17-29 doi:10.1093/esr/jcn081

Böröcz J, Southworth C (1996) Decomposing the intellectuals' class power: Conversion of cultural capital to income, Hungary, 1986. Social Forces 74(3):797-822

Choi NG, Burr JA, Mutchler JE, Caro FG (2007) Formal and Informal Volunteer Activity and Spousal Caregiving Among Older Adults. Research on Aging 29(2):99-124

Choi NG, Chou RJA (2010) Time and money volunteering among older adults: the relationship between past and current volunteering and correlates of change and stability. Ageing & Society 30:559-581 doi:10.1017/s0144686x0999064x

Corten R, Dronkers J (2006) School Achievement of Pupils From the Lower Strata in Public, Private Government-Dependent and Private Government-Independent Schools: A cross-national test of the Coleman-Hoffer thesis. Educational Research and Evaluation 12(2)

De Graaf ND, De Graaf PM (2002) Formal and popular dimensions of cultural capital: Effects on children's educational attainment. Netherlands' Journal of Social Sciences 38(2):167-186

De Graaf ND, De Graaf PM, Kraaykamp G (2000) Parental cultural capital and educational attainment in the Netherlands: A refinement of the cultural capital perspective. Sociology of Education 73(2):92-111

De Graaf PM (1986) The Impact of Financial and Cultural Resources on Educational Attainment in the Netherlands. Sociology of Education 59(4):237-246

DiMaggio P (1982) Cultural Capital and School Success: The Impact of Status Culture Participation on the Grades of U.S. High School Students. American Sociological Review 47(2):189-201

DiMaggio P, Mohr J (1985) Cultural Capital, Educational Attainment, and Marital Selection. The American Journal of Sociology 90(6):1231-1261

DiMaggio P, Mukhtar T (2004) Arts participation as cultural capital in the United States, 1982-2002: Signs of decline? Poetics 32(2):169-194 doi:10.1016/j.poetic.2004.02.005

Driessen G, Smit F (2007) Effects of immigrant parents' participation in society on their children's school performance. Acta Sociologica 50(1):39-56 doi:10.1177/0001699307074882

Dumais S (2002) Cultural Capital, Gender, and School Success: The Role of Habitus. Sociology of Education 75(1):44-68

Dumais S (2006a) Early childhood cultural capital, parental habitus, and teachers' perceptions. Poetics 34(2):83-107 doi:10.1016/j.poetic.2005.09.003

Dumais S (2006b) Elementary school students' extracurricular activities: The effects of participation on achievement and teachers' evaluations. Sociological Spectrum 26(2):117-147 doi:10.1080/02732170500444593

Dumais S, Ward A (2010) Cultural capital and first-generation college success. Poetics 38(3):245-265 doi:10.1016/j.poetic.2009.11.011

Egerton M (1997) Occupational inheritance: The role of cultural capital and gender. Work Employment and Society 11(2):263-282

Eitle TM, Eitle DJ (2002) Race, Cultural Capital, and the Educational Effects of Participation in Sports. Sociology of Education 75(2):123-146

Erickson BH (1991) What Is Good Taste Good For. Can Rev Soc Anthrop 28(2):255-278

Erickson BH (1996) Culture, Class, and Connections. The American Journal of Sociology 102(1):217-251

Flere S, Krajnc MT, Klanjsek R, Musil B, Kirbis A (2010) Cultural capital and intellectual ability as predictors of scholastic achievement: a study of Slovenian secondary school students. British Journal of Sociology of Education 31(1):47-58 doi:10.1080/01425690903385428

Fujimoto K (2004) Feminine capital: The forms of capital in the female labor market in Japan. Sociological Quarterly 45(1):91-111

Georg W (2004) Cultural Capital and Social Inequality in the Life Course. European Sociological Review 20(4):333-344

Houtman D (2000) The working class and the welfare state support for economic redistribution, tolerance for nonconformity, and the conditionality of solidarity with the unemployed. Netherlands Journal of Social Sciences 36(1):37-55

Jæger MM (2009) Equal Access but Unequal Outcomes: Cultural Capital and Educational Choice in a Meritocratic Society. Social Forces 87(4):1943-1971

Jagodzinski W (2010) Economic, Social, and Cultural Determinants of Life Satisfaction: Are there Differences Between Asia and Europe? Social Indicators Research 97(1):85-104 doi:10.1007/s11205-009-9555-1

Johnson JH, Bienenstock EJ, Stoloff JA (1995) An empirical test of the cultural capital hypothesis. Review of Black Political Economy 23(4):7-27

Johnson JH, Farrell WC, Stoloff JA (2000) An empirical assessment of four perspectives on the declining fortunes of the African-American male. Urban Affairs Review 35(5):695-716

Jonsson JO (1987) Class Origin, Cultural Origin, and Educational Attainment: The Case of Sweden. European Sociological Review 3(3):229-242

Kalmijn M, Kraaykamp G (1996) Race, Cultural Capital, and Schooling: An Analysis of Trends in the United States. Sociology of Education 69(1):22-34

Kanaan MN, Afifi RA (2010) Gender differences in determinants of weight-control behaviours among adolescents in Beirut. Public Health Nutr 13(1):71-81. Epub 2009 Feb 26.

Karen D (1991) "Achievement" and "ascription" in admission to an elite college: A political-organizational analysis. Sociological Forum 6(2):349-380

Katsillis J, Rubinson R (1990) Cultural Capital, Student Achievement, and Educational Reproduction: The Case of Greece. American Sociological Review 55(2):270-279

Katz-Gerro T, Sullivan O (2010) Voracious Cultural Consumption The intertwining of gender and social status. Time & Society 19(2):193-219 doi:10.1177/0961463x09354422

Kaufman J, Gabler J (2004) Cultural capital and the extracurricular activities of girls and boys in the college attainment process. Poetics 32(2):145-168 doi:10.1016/j.poetic.2004.02.001

Kay FM, Hagan J (1998) Raising the bar: The gender stratification of law-firm capital. American Sociological Review 63(5):728-743

Khawaja M, Barazi R, Linos N (2007) Maternal cultural participation and child health status in a Middle Eastern context: Evidence from an urban health study. Child: Care, Health and Development 33(2):117-125

Khawaja M, Mowafi M (2006) Cultural capital and self-rated health in low income women evidence from the urban health study, Beirut, Lebanon. Journal of Urban Health 83(3):444-458 doi:10.1007/s11524-006-9051-8

Khawaja M, Mowafi M (2007) Types of cultural capital and self-rated health among disadvantaged women in outer Beirut, Lebanon. Scandinavian Journal of Public Health 35(5):475-480 doi:10.1080/14034940701256958

Kim J, Kang JH, Lee MA, Lee Y (2007) Volunteering among older people in Korea. Journals of Gerontology 62(1):S69-S73

Kim PH, Aldrich HE, Keister LA (2006) Access (not) denied: The impact of financial, human, and cultural capital on entrepreneurial entryin the United States. Small Business Economics 27(1):5-22 doi:10.1007/s11187-006-0007-x

Kim S, Kim H (2009) Does Cultural Capital Matter?: Cultural Divide and Quality of Life. Social Indicators Research 93(2):295-313 doi:10.1007/s11205-008-9318-4

Kingston P, Hubbard R, Lapp B, Schroeder P, Wilson J (2003) Why education matters. Sociology of Education 76(1):53-70

Lamb S (1989) Cultural Consumption and the Educational Plans of Australian Secondary School Students. Sociology of Education 62(2):95-108

Lampard R (2007) Is social mobility an echo of educational mobility? Parents' educations and occupations and their children's occupational attainment. Sociological Research Online 12(5)

Lee EM, Kao G (2009) Less bang for the buck? Cultural capital and immigrant status effects on kindergarten academic outcomes. Poetics 37(3):201-226 doi:10.1016/j.poetic.2009.02.001

Lee Y (2009) Early motherhood and harsh parenting: The role of human, social, and cultural capital. Child Abuse & Neglect 33(9):625-637 doi:10.1016/j.chiabu.2009.02.007

Lewicka M (2005) Ways to make people active: The role of place attachment, cultural capital, and neighborhood ties. Journal of Environmental Psychology 25(4):381-395 doi:10.1016/j.jenvp.2005.10.004

Lizardo O (2005) Can cultural capital theory be reconsidered in the light of world polity institutionalism? Evidence from Spain. Poetics 33(2):81-110 doi:10.1016/j.poetic.2005.02.001

Lizardo O (2006) How Cultural Tastes Shape Personal Networks. American Sociological Review 71(5):778-807

Marjoribanks K, Kwok Y (1998) Family capital and Hong Kong adolescents academic achievement. Psychological Reports 83(1):99-105

Mehus I (2005) Distinction through Sport Consumption: Spectators of Soccer, Basketball, and Ski-jumping. International Review for the Sociology of Sport 40(3):321-333

Mickelson RA, Greene AD (2006) Connecting Pieces of the Puzzle: Gender Differences in Black Middle School Students' Achievement. Journal of Negro Education 75(1):34-48

Mitchell BA (1994) Family structure and leaving the nest: A social resource perspective. Sociological Perspectives 37(4):651-671

Myrberg E, Rosen M (2006) Reading achievement and social selection in independent schools in Sweden: Results from IEA PIRLS 2001. Scandinavian Journal of Educational Research 50(2):185-205

Myrberg E, Rosen M (2008) A path model with mediating factors of parents' education on students' reading achievement in seven countries. Educational Research and Evaluation 14(6):507-520

Myrberg E, Rosen M (2009) Direct and indirect effects of parents' education on reading achievement among third graders in Sweden. British Journal of Educational Psychology 79:695-711 doi:10.1348/000709909x453031

Noble J, Davies P (2009) Cultural capital as an explanation of variation in participation in higher education. British Journal of Sociology of Education 30(5):591-605 doi:10.1080/01425690903101098

Nora A (2004) The role of habitus and cultural capital in choosing a college, transitioning from high school to higher education, and persisting in college among minority and nonminority students. Journal of Hispanic Higher Education 3(2):180-208

Oates GL (2009) An empirical test of five prominent explanations for the black-white academic performance gap. Social Psychology of Education 12(4):415-441

Okun MA, Michel J (2006) Sense of community and being a volunteer among the young-old. Journal of Applied Gerontology 25(2):173-188

Orr AJ (2003) Black-white differences in achievement: The importance of wealth. Sociology of Education 76(4):281-304

Osinsky P, Mueller CW (2004) Professional Commitment of Russian Provincial Specialists. Work and Occupations 31(2):193-224

Parboteeah KP, Cullen JB, Lim L (2004) Formal volunteering: a cross-national test. Journal of World Business 39(4):431-441 doi:10.1016/j.jwb.2004.08.007

Pearce RR, Lin Z (2007) Chinese American post-secondary achievement and attainment: a cultural and structural analysis. Educational Review 59(1):19-36 doi:10.1080/00131910600796827

Pedersen W (1996) Working-class boys at the margins: Ethnic prejudice, cultural capital, and gender. Acta Sociologica 39(3):257-279

Pellerin LA, Stearns E (2001) Status honor and the valuing of cultural and material capital. Poetics 29(1):1-24

Perreira KM, Harris KM, Lee D (2006) Making it in America: High school completion by immigrant and native youth. Demography 43(3):511-536

Pettit B (1999) Cultural capital and residential mobility: A model of impersistence in place. Poetics 26(3):177-199

Pinheiro DL, Dowd TJ (2009) All that jazz: The success of jazz musicians in three metropolitan areas. Poetics 37(5):490-506 doi:10.1016/j.poetic.2009.09.007

Powell B, Steelman LC, Carini RM (2006) Advancing age, advantaged youth: Parental age and the transmission of resources to children. Social Forces 84(3):1359-1390

Prieur A, Rosenlund L, Skjott-Larsen J (2008) Cultural capital today - A case study from Denmark. Poetics 36(1):45-71 doi:10.1016/j.poetic.2008.02.008

Robinson RV, Garnier MA (1985) Class Reproduction Among Men and Women in France: Reproduction Theory on Its Home Ground. The American Journal of Sociology 91(2):250-280

Roose H, Vandenhaute D (2010) Art world on stage: Analyzing the social logic of literary realities and aesthetic dispositions in the contemporary Flemish theatre world. Poetics 38(1):90-108 doi:10.1016/j.poetic.2009.11.001

Roscigno VJ, Ainsworth-Darnell JW (1999) Race, Cultural Capital, and Educational Resources: Persistent Inequalities and Achievement Returns. Sociology of Education 72(3):158-178

Roscigno VJ, Tomaskovic-Devey D, Crowley M (2006) Education and the Inequalities of Place. Social Forces 84(4):2121-2145

Salisbury MH, Umbach PD, Paulsen MB, Pascarella ET (2009) Going Global: Understanding the Choice Process of the Intent to Study Abroad. Research in Higher Education 50(2):119-143 doi:10.1007/s11162-008-9111-x

Scherger S, Savage M (2010) Cultural transmission, educational attainment and social mobility. Sociological Review 58(3):406-428 doi:10.1111/j.1467-954X.2010.01927.x

Solhaug T (2009) Two configurations for accessing classroom computers: differential impact on students' critical reflections and their empowerment. Journal of Computer Assisted Learning 25(5):411-422 doi:10.1111/j.1365-2729.2009.00318.x

Stempel C (2005) Adult Participation Sports as Cultural Capital: A Test of Bourdieu's Theory of the Field of Sports. International Review for the Sociology of Sport 40(4):pp

Strayhorn TL (2010) When Race and Gender Collide: Social and Cultural Capital's Influence on the Academic Achievement of African American and Latino Males. Review of Higher Education 33(3):307-332

Sullivan A (2001) Cultural capital and educational attainment. Sociology 35(4):893-912

Tang FY (2006) What resources are needed for volunteerism? A life course perspective. Journal of Applied Gerontology 25(5):375-390 doi:10.1177/0733464806292858

Tanner J, Asbridge M, Wortley S (2008) Our favourite melodies: Musical consumption and teenage lifestyles. British Journal of Sociology 59(1):117-144

Tonmyr L, Jamieson E, Mery LS, MacMillan HL (2006) Child Abuse and Disability in an Ontario Community Sample: Does Social Capital Matter? The International Journal of Mental Health Promotion 8(2):23-30

Tramonte L, Willms JD (2010) Cultural capital and its effects on education outcomes. Economics of Education Review 29(2):200-213 doi:10.1016/j.econedurev.2009.06.003

Trienekens S (2002) 'Colourful' distinction: The role of ethnicity and ethnic orientation in cultural consumption. Poetics 30(4):281-298

Turmo A (2004) Scientific literacy and socio-economic background among 15-year-olds--A Nordic perspective. Scandinavian Journal of Educational Research 48(3):287-305

Van de Werfhorst HG, Hofstede S (2007) Cultural capital or relative risk aversion? Two mechanisms for educational inequality compared. British Journal of Sociology 58(3):391-415

Van de Werfhorst HG, Kraaykamp G (2001) Four Field-Related Educational Resources and Their Impact on Labor, Consumption, and Sociopolitical Orientation. Sociology of Education 74(4):296-317

Van de Werfhorst HG, Sullivan A, Cheung SY (2003) Social Class, Ability and Choice of Subject in Secondary and Tertiary Education in Britain. British Educational Research Journal 29(1):41-62

Van der Velden RKW (1996) Family socialization and educational attainment: Evidence from a Dutch cohort. Netherlands Journal of Social Sciences 32(2):90-108

Van Wel F, Couwenbergh-Soeterboek N, Couwenbergh C, Ter Bogt T, Raaijmakers Q (2006) Ethnicity, youth cultural participation, and cultural reproduction in the Netherlands. Poetics 34(1):65-82 doi:10.1016/j.poetic.2005.06.001

Vryonides M (2007) Social and cultural capital in educational research: issues of operationalisation and measurement. British Educational Research Journal 33(6):867-885 doi:10.1080/01411920701657009

Wildhagen T (2009) Why does cultural capital matter for high school academic performance? An empirical assessment of teacher-selection and self-selection mechanisms as explanations of the cultural capital effect. The Sociological Quarterly 50(1):173-200

Wilson J, Musick M (1997) Who cares? Toward an integrated theory of volunteer work. American Sociological Review 62(5):694-713

Wu Y (2008) Cultural capital, the state, and educational inequality in China, 1949-1996. Sociological Perspectives 51(1):201-227

Wuthnow R (2000) How religious groups promote forgiving: A national study. Journal for the Scientific Study of Religion 39(2):125-139

Yamamoto Y, Brinton MC (2010) Cultural Capital in East Asian Educational Systems: The Case of Japan. Sociology of Education 83(1):67-83 doi:10.1177/0038040709356567

Zarycki T (2007) Cultural capital and the accessibility of higher education. Russian Education and Society 49(7):41-72 doi:10.2753/res1060-9393490703

Zimdars A, Sullivan A, Heath A (2009) Elite Higher Education Admissions in the Arts and Sciences: Is Cultural Capital the Key? Sociology-the Journal of the British Sociological Association 43(4):648-666 doi:10.1177/0038038509105413
