# Supplementary material for: Bourdieu’s Cultural Capital in Relation to Food Choices: A Systematic Review of Cultural Capital Indicators and an Empirical Proof of Concept
Source: PLoS One. 2015 Aug 5;10(8):e0130695. doi: 10.1371/journal.pone.0130695 (PMC4526463; doi:10.1371/journal.pone.0130695)
Supplement: S2 Tables — (DOC) [file pone.0130695.s004.doc]

**Web-appendix 2: Results of factor analyses**

**Factor analysis for cooking skills**

| **Communalities** | | |
| --- | --- | --- |
|  | Initial | Extraction |
| skills_vegs | 1,000 | ,787 |
| skills_fish | 1,000 | ,616 |
| skills_norecipy | 1,000 | ,711 |
| Extraction Method: Principal Component Analysis. | | |

| **Total Variance Explained** | | | | | | |
| --- | --- | --- | --- | --- | --- | --- |
| Component | Initial Eigenvalues | | | Extraction Sums of Squared Loadings | | |
|  | Total | % of Variance | Cumulative % | Total | % of Variance | Cumulative % |
| 1 | 2,114 | 70,458 | 70,458 | 2,114 | 70,458 | 70,458 |
| 2 | ,560 | 18,661 | 89,120 |  |  |  |
| 3 | ,326 | 10,880 | 100,000 |  |  |  |
| Extraction Method: Principal Component Analysis. | | | | | | |

| **Component Matrix^a^** | |
| --- | --- |
|  | Component |
|  | 1 |
| skills_vegs | ,887 |
| skills_fish | ,785 |
| skills_norecipy | ,843 |
| Extraction Method: Principal Component Analysis. | |
| a. 1 components extracted. | |

**Factor analysis for grocery shopping skills**

| **Communalities** | | |
| --- | --- | --- |
|  | Initial | Extraction |
| skills_shop | 1,000 | ,689 |
| skills_shoplist | 1,000 | ,689 |
| Extraction Method: Principal Component Analysis. | | |

| **Total Variance Explained** | | | | | | |
| --- | --- | --- | --- | --- | --- | --- |
| Component | Initial Eigenvalues | | | Extraction Sums of Squared Loadings | | |
|  | Total | % of Variance | Cumulative % | Total | % of Variance | Cumulative % |
| 1 | 1,378 | 68,878 | 68,878 | 1,378 | 68,878 | 68,878 |
| 2 | ,622 | 31,122 | 100,000 |  |  |  |
| Extraction Method: Principal Component Analysis. | | | | | | |

| **Component Matrix^a^** | |
| --- | --- |
|  | Component |
|  | 1 |
| skills_shop | ,830 |
| skills_shoplist | ,830 |
| Extraction Method: Principal Component Analysis. | |
| a. 1 components extracted. | |

**Factor analysis for food information skills**

| **Communalities** | | |
| --- | --- | --- |
|  | Initial | Extraction |
| skills_read | 1,000 | ,781 |
| skills_decis | 1,000 | ,798 |
| skills_web | 1,000 | ,439 |
| skills_recipe | 1,000 | ,724 |
| Extraction Method: Principal Component Analysis. | | |

| **Total Variance Explained** | | | | | | | | | |
| --- | --- | --- | --- | --- | --- | --- | --- | --- | --- |
| Component | Initial Eigenvalues | | | Extraction Sums of Squared Loadings | | | Rotation Sums of Squared Loadings | | |
|  | Total | % of Variance | Cumulative % | Total | % of Variance | Cumulative % | Total | % of Variance | Cumulative % |
| 1 | 1,732 | 43,290 | 43,290 | 1,732 | 43,290 | 43,290 | 1,610 | 40,246 | 40,246 |
| 2 | 1,010 | 25,251 | 68,541 | 1,010 | 25,251 | 68,541 | 1,132 | 28,295 | 68,541 |
| 3 | ,861 | 21,537 | 90,078 |  |  |  |  |  |  |
| 4 | ,397 | 9,922 | 100,000 |  |  |  |  |  |  |
| Extraction Method: Principal Component Analysis. | | | | | | | | | |

| **Component Matrix^a^** | | |
| --- | --- | --- |
|  | Component | |
|  | 1 | 2 |
| skills_read | ,846 | -,255 |
| skills_decis | ,844 | -,293 |
| skills_web | ,458 | ,479 |
| skills_recipe | ,307 | ,794 |
| Extraction Method: Principal Component Analysis. | | |
| a. 2 components extracted. | | |

**Factor analysis for all cultural capital items skills (with sum score for food knowledge, and without general values items)**

| **Component Matrix^a^** | | | | | | |
| --- | --- | --- | --- | --- | --- | --- |
|  | Component | | | | | |
|  | 1 | 2 | 3 | 4 | 5 | 6 |
| educ_vader_v9 | ,219 | ,842 | -,294 | -,092 | -,125 | -,019 |
| educ_moeder_v9 | ,212 | ,848 | -,270 | -,103 | -,109 | -,008 |
| educ_partner_v9 | ,157 | ,531 | -,166 | ,001 | -,060 | -,009 |
| Oven_v45_9 | ,781 | -,218 | -,294 | -,013 | -,018 | -,050 |
| Scales_v45_9 | ,699 | -,200 | -,420 | -,102 | ,054 | -,074 |
| Cookb_v45_9 | ,734 | -,209 | -,369 | -,104 | ,019 | -,067 |
| Juicer_v45_9 | ,742 | -,232 | -,371 | -,091 | ,022 | -,050 |
| knife_v45_9 | ,760 | -,249 | -,377 | -,057 | -,014 | -,041 |
| skills_fish9 | ,467 | ,062 | ,425 | -,525 | ,120 | ,072 |
| skills_norecipy9 | ,478 | ,042 | ,432 | -,548 | ,229 | ,103 |
| skills_vegs9 | ,423 | ,060 | ,462 | -,524 | ,038 | -,059 |
| skills_read9 | ,485 | ,069 | ,376 | ,295 | -,330 | -,180 |
| skills_decis9 | ,519 | ,050 | ,355 | ,248 | -,339 | -,284 |
| skills_web9 | ,671 | -,022 | ,258 | ,215 | -,151 | ,044 |
| skills_recipe9 | ,521 | ,040 | ,372 | ,249 | -,352 | -,220 |
| skills_shop9 | ,393 | ,074 | ,117 | ,185 | -,106 | ,697 |
| skills_shoplist9 | ,479 | -,016 | ,022 | ,232 | -,145 | ,563 |
| food_know2 | -,178 | -,366 | ,089 | ,076 | -,054 | ,053 |
| v60_eat_yn9 | ,400 | ,177 | ,148 | ,394 | ,702 | -,089 |
| v60_eatout_yn9 | ,408 | ,197 | ,169 | ,407 | ,679 | -,051 |
| Extraction Method: Principal Component Analysis. | | | | | | |
| a. 6 components extracted. | | | | | | |
