# Supplementary material for: Bourdieu’s Cultural Capital in Relation to Food Choices: A Systematic Review of Cultural Capital Indicators and an Empirical Proof of Concept
Source: PLoS One. 2015 Aug 5;10(8):e0130695. doi: 10.1371/journal.pone.0130695 (PMC4526463; doi:10.1371/journal.pone.0130695)
Supplement: S3 Tables — (DOC) [file pone.0130695.s005.doc]

**S3:** Odds ratios for unhealthy food choices by specific cultural capital items (adjusted for age, sex, and socioeconomic position^a^)

|  | **N** **(%)**  (n=2952) | **OR for eating more unhealthy than healthy bread products**  (n=2781)^b^ | | | **OR for eating more unhealthy than healthy meat products**  (n=2783) ^b^ | | | **OR for eating more unhealthy than healthy snacks**  (n=2735) ^b^ | | |
| --- | --- | --- | --- | --- | --- | --- | --- | --- | --- | --- |
|  |  | **OR** | **(95% CI)** | **p** | **OR** | **(95% CI)** | **p** | **OR** | **(95% CI)** | **p** |
|  |  |  |  |  |  |  |  |  |  |  |
| Items for family institutionalised cultural capital |  |  |  |  |  |  |  |  |  |  |
| Educational level of father |  |  |  | .001 |  |  | .178 |  |  | .124 |
| 1 Low | 20.1 | 3.03 | (1.68-5.48) |  | 1.27 | (0.96-1.70) |  | 1.73 | (1.11-2.69) |  |
| 2 | 23.3 | 1.62 | (0.90-2.91) |  | 1.03 | (0.79-1.35) |  | 1.33 | (0.88-2.00) |  |
| 3 | 15.1 | 1.48 | (0.79-2.79) |  | 0.92 | (0.69-1.23) |  | 1.24 | (0.80-1.94) |  |
| 4 High | 14.8 | 1.00 |  |  | 1.00 |  |  | 1.00 |  |  |
| Missing | 26.7 | 2.48 | (1.35-4.56) |  | 1.04 | (0.78-1.39) |  | 1.66 | (1.06-2.60) |  |
| Educational level of mother |  |  |  | .048 |  |  | .242 |  |  | .160 |
| 1 Low | 26.3 | 4.41 | (1.46-13.31) |  | 1.36 | (0.91-2.04) |  | 2.25 | (1.14-4.46) |  |
| 2 | 31.9 | 2.94 | (0.99-8.72) |  | 1.14 | (0.78-1.68) |  | 2.03 | (1.06-3.90) |  |
| 3 | 10.6 | 2.83 | (0.89-8.99) |  | 0.99 | (0.64-1.53) |  | 1.73 | (0.84-3.55) |  |
| 4 High | 5.1 | 1.00 |  |  | 1.00 |  |  | 1.00 |  |  |
| Missing | 26.1 | 3.91 | (1.27-12.08) |  | 1.21 | (0.80-1.84) |  | 2.38 | (1.17-4.82) |  |
| Educational level of partner |  |  |  | .0000 |  |  | .026 |  |  | .094 |
| 1 Low | 4.0 | 4.71 | (2.13-10.44) |  | 0.99 | (0.62-1.58) |  | 0.99 | (0.41-2.40) |  |
| 2 | 22.9 | 2.62 | (1.55-4.43) |  | 1.33 | (1.03-1.71) |  | 1.51 | (1.02-2.23) |  |
| 3 | 24.4 | 1.93 | (1.20-3.09) |  | 1.09 | (0.87-1.35) |  | 1.53 | (1.10-2.15) |  |
| 4 High | 29.5 | 1.00 |  |  | 1.00 |  |  | 1.00 |  |  |
| Missing | 19.1 | 2.71 | (1.63-4.52) |  | 0.88 | (0.68-1.13) |  | 1.21 | 0.80-1.82) |  |
| Items for objectivised cultural capital |  |  |  |  |  |  |  |  |  |  |
| Possession of oven |  |  |  | .035 |  |  | .047 |  |  | .093 |
| No | 3.8 | 1.44 | (0.70-2.99) |  | 1.39 | (0.91-2.11) |  | 0.67 | (0.30-1.50) |  |
| Yes | 94.5 | 1.00 |  |  | 1.00 |  |  | 1.00 |  |  |
| Missing | 1.7 | 3.10 | (1.26-7.60) |  | 2.10 | (1.02-4.32) |  | 2.31 | (0.98-5.48) |  |
| Possession of scales |  |  |  | .004 |  |  | .111 |  |  | .043 |
| No | 17.4 | 1.58 | (1.11-2.25) |  | 1.13 | (0.92-1.38) |  | 0.94 | (0.68-1.29) |  |
| Yes | 79.9 | 1.00 |  |  | 1.00 |  |  | 1.00 |  |  |
| Missing | 2.7 | 2.80 | (1.23-6.37) |  | 1.72 | (0.97-3.06) |  | 2.60 | (1.20-5.61) |  |
| Posession of cookbook |  |  |  | .0000 |  |  | .118 |  |  | .039 |
| No | 9.6 | 2.61 | (1.72-3.97) |  | 0.94 | (0.71-1.25) |  | 0.70 | (0.44-1.13) |  |
| Yes | 87.4 | 1.00 |  |  | 1.00 |  |  | 1.00 |  |  |
| Missing | 3.0 | 4.20 | (2.03-8.72) |  | 1.68 | (1.00-2.83) |  | 2.00 | (0.99-4.04) |  |
| Posession of juicer |  |  |  | .027 |  |  | .002 |  |  | .023 |
| No | 23.8 | 1.49 | (1.08-2.06) |  | 1.21 | (1.01-1.46) |  | 1.35 | (1.04-1.76) |  |
| Yes | 73.5 | 1.00 |  |  | 1.00 |  |  | 1.00 |  |  |
| Missing | 2.7 | 1.96 | (0.80-4.76) |  | 2.31 | (1.33-4.03) |  | 2.06 | (0.96-4.46) |  |
| Posession of set of knives |  |  |  | .141 |  |  | .004 |  |  | .229 |
| No | 12.1 | 1.20 | (0.77-1.87) |  | 0.83 | (0.65-1.07) |  | 1.09 | (0.76-1.59) |  |
| Yes | 85.4 | 1.00 |  |  | 1.00 |  |  | 1.00 |  |  |
| Missing | 2.5 | 2.19 | (0.97-4.96) |  | 2.39 | (1.32-4.34) |  | 1.92 | (0.90-4.09) |  |
|  |  |  |  |  |  |  |  |  |  |  |
| Items for incorporated cultural capital |  |  |  |  |  |  |  |  |  |  |
| Cooking skills |  |  |  |  |  |  |  |  |  |  |
| Know how to prepare fish |  |  |  | .002 |  |  | .000 |  |  | .000 |
| Disagree | 31.1 | 1.75 | (1.29-2.37) |  | 2.14 | (1.80-2.54) |  | 2.00 | (1.56-2.56) |  |
| Agree | 66.5 | 1.00 |  |  | 1.00 |  |  | 1.00 |  |  |
| Missing | 2.4 | 1.31 | (0.39-4.39) |  | 2.14 | (1.18-3.89) |  | 0.48 | (0.10-2.21) |  |
| Can prepare meals without recipe |  |  |  | .086 |  |  | .041 |  |  | .030 |
| Disagree | 28.5 | 1.35 | (0.98-1.86) |  | 1.26 | (1.05-1.50) |  | 1.38 | (1.06-1.78) |  |
| Agree | 70.4 | 1.00 |  |  | 1.00 |  |  | 1.00 |  |  |
| Missing | 1.1 | 2.89 | (0.67-12.55) |  | 1.36 | (0.53-3.50) |  | 0.20 | (0.01-5.28) |  |
| Know several ways to prepare vegetables |  |  |  | .010 |  |  | .000 |  |  | .000 |
| Disagree | 29.2 | 1.61 | (1.17-2.20) |  | 1.57 | (1.32-1.88) |  | 2.28 | (1.77-2.93) |  |
| Agree | 67.8 | 1.00 |  |  | 1.00 |  |  | 1.00 |  |  |
| Missing | 3.0 | 0.82 | (0.25-2.68) |  | 1.28 | (0.77-2.13) |  | 0.30 | (0.06-1.48) |  |
| Nutrition information skills |  |  |  |  |  |  |  |  |  |  |
| Read nutrition information on food packages |  |  |  | .222 |  |  | .000 |  |  | .006 |
| Sometimes- (almost) never | 62.2 | 1.31 | (0.93-1.83) |  | 1.42 | (1.20-1.68) |  | 1.57 | (1.19-2.07) |  |
| Always-usually | 34.7 | 1.00 |  |  | 1.00 |  |  | 1.00 |  |  |
| Missing | 3.1 | 1.67 | (0.72-3.90) |  | 1.55 | (0.93-2.59) |  | 1.19 | (0.52-2.74) |  |
| Use information on food packages for food decisions |  |  |  | .145 |  |  | .000 |  |  | .074 |
| Sometimes- (almost) never | 69.7 | 1.45 | (0.98-2.13) |  | 1.52 | (1.26-1.84) |  | 1.43 | (1.05-1.95) |  |
| Always-usually | 25.1 | 1.00 |  |  | 1.00 |  |  | 1.00 |  |  |
| Missing | 5.2 | 1.63 | (0.79-3.35) |  | 1.52 | (1.03-2.27) |  | 1.40 | (0.75-2.61) |  |
| Look up information about food on the internet |  |  |  | .049 |  |  | .075 |  |  | .439 |
| Sometimes- (almost) never | 95.8 | 0.92 | (0.38-2.25) |  | 1.38 | (0.83-2.31) |  | 1.11 | (0.50-2.46) |  |
| Always-usually | 2.6 | 1.00 |  |  | 1.00 |  |  | 1.00 |  |  |
| Missing | 1.6 | 3.01 | (0.84-10.82) |  | 2.87 | (1.16-7.13) |  | 2.14 | (0.59-7.72) |  |
| Use recipes from cookery books, internet or magazines |  |  |  | .343 |  |  | .000 |  |  | .057 |
| Sometimes- (almost) never | 30.6 | 1.19 | (0.73-1.94) |  | 1.64 | (1.28-2.11) |  | 1.44 | (0.95-2.16) |  |
| Always-usually | 67.5 | 1.00 |  |  | 1.00 |  |  | 1.00 |  |  |
| Missing | 1.9 | 1.77 | (0.82-3.84) |  | 1.93 | (1.24-3.01) |  | 2.20 | (1.14-4.24) |  |
| Shopping skills |  |  |  |  |  |  |  |  |  |  |
| Before going shopping, I make a list of what I need |  |  |  | .000 |  |  | .456 |  |  | .731 |
| Sometimes- (almost) never | 30.6 | 1.75 | (1.28-2.38) |  | 1.10 | (0.93-1.30) |  | 1.10 | (0.85-1.41) |  |
| Always-usually | 67.5 | 1.00 |  |  | 1.00 |  |  | 1.00 |  |  |
| Missing | 1.9 | 5.02 | (2.26-11.19) |  | 1.26 | (0.66-2.39) |  | 1.22 | (0.44-3.37) |  |
| I decide what to buy when I am at the shop |  |  |  | .138 |  |  | .006 |  |  | .958 |
| Always-sometimes | 52.2 | 1.18 | (0.86-1.60) |  | 1.25 | (1.06-1.46) |  | 0.97 | (0.756-1.23) |  |
| (Almost) never | 43.4 | 1.00 |  |  | 1.00 |  |  | 1.00 |  |  |
| Missing | 4.4 | 2.03 | (0.98-4.26) |  | 1.65 | (1.07-2.53) |  | 1.03 | (0.48-2.22) |  |
| Food knowledge |  |  |  | .000 |  |  | .001 |  |  | .000 |
| Low-moderate | 43.4 | 1.59 | (1.15-2.20) |  | 1.34 | (1.13-1.58) |  | 1.35 | (1.04-1.75) |  |
| Good-excellent | 52.5 | 1.00 |  |  | 1.00 |  |  | 1.00 |  |  |
| Missing | 4.2 | 3.75 | (2.04-6.89) |  | 1.71 | (1.11-2.62) |  | 3.33 | (1.95-5.66) |  |
| Food participation |  |  |  |  |  |  |  |  |  |  |
| Meeting in your or other people’s home to have dinner at least once a month |  |  |  | .045 |  |  | .000 |  |  | .690 |
| No | 19.0 | 1.57 | (1.10-2.25) |  | 1.71 | (1.40-2.09) |  | 1.14 | (0.84-1.56) |  |
| Yes | 79.0 | 1.00 |  |  | 1.00 |  |  | 1.00 |  |  |
| Missing | 2.1 | 1.47 | (0.45-4.85) |  | 1.00 | (0.51-1.96) |  | 1.18 | (0.40-3.44) |  |
| Meeting with people in public place to have food at least once a month |  |  |  | .099 |  |  | .002 |  |  | .457 |
| No | 28.5 | 1.41 | (1.03-1.93) |  | 1.36 | (1.14-1.61) |  | 1.18 | (0.91-1.54) |  |
| Yes | 68.9 | 1.00 |  |  | 1.00 |  |  | 1.00 |  |  |
| Missing | 2.6 | 0.97 | (0.29-3.21) |  | 0.85 | (0.48-1.52) |  | 1.05 | (0.41-2.70) |  |
|  |  |  |  |  |  |  |  |  |  |  |

^a^ Socioeconomic position was measured by the respondent’s highest attained educational level.
^b^ Varying sample sizes due to different numbers of missing values on the food choice outcomes.
